# The association between autism and psychosis and the tools used to measure it: An updated systematic review and meta‐analysis

**DOI:** 10.1111/bjc.70020

**Published:** 2025-12-16

**Authors:** Michael R. Miles, Dennis Golm, Emma Palmer‐Cooper

**Affiliations:** ^1^ Centre for Innovation in Mental Health, School of Psychology, Faculty of Environmental and Life Sciences University of Southampton Southampton UK

**Keywords:** Autism, Methodology, Psychopathology/Psychological Disorders, Meta analyses/systematic reviews, Psychopathology/Psychological Disorders, Personality disorders, Psychopathology/Psychological Disorders, Psychosis/schizophrenia

## Abstract

**Objectives:**

Autistic individuals are at increased risk of psychotic experiences and being diagnosed with psychotic disorders. This association may result from methodological issues, including the misinterpretation of psychosis questionnaires by autistic individuals and clinicians' difficulty distinguishing between the conditions.

**Design:**

This meta‐analysis aimed to review this association and examine whether it is moderated by the assessment measures used.

**Methods:**

Systematic searches were conducted in PsycINFO, MEDLINE, CINAHL, Embase and Web of Science. Included studies required autism and psychosis‐spectrum measurements, co‐morbidity data, adult participants and quantitative data. Quality and risk of bias were assessed using the AXIS Critical Appraisal of Cross‐Sectional Studies tool. Analyses examined correlations, odds ratios and Cohen's *d* as effects.

**Results:**

Sixty‐three papers (*N* = 6,903,960) were included. Associations were found between autistic and overall (*r* = .435, *p* < .0001), positive (*r* = .274, *p* < .0001), negative (*r* = .506, *p* < .0001) and disorganized (*r* = .366, *p* < .0001) psychosis‐spectrum traits. Individuals with one condition had an increased risk of being diagnosed with the other (OR = 7.03, *p* < .001) and scored higher on trait measures of the other (*d* = 1.187, *p* < .0001).

**Conclusions:**

These meta‐analyses evidence a strong association between autism and the psychosis spectrum, at both trait and diagnostic levels. Negative psychosis‐spectrum traits were most strongly linked with autistic traits, while measures of positive traits showed weaker correlations, suggesting overlaps in expression and measurement. High heterogeneity and inconsistent reporting, however, hinder the certainty of conclusions, and research is required to better understand this overlap.

**Practitioner points**

1. Autistic individuals are significantly more likely to receive a psychotic disorder diagnosis and score higher on psychosis‐spectrum traits.
2. At a trait level, autistic traits are most strongly associated with negative psychosis‐spectrum traits, with weaker associations being found for positive traits.
3. Assessments of psychotic disorders in autistic individuals should use tools that require concrete examples for psychotic phenomena, and the developmental history must be taken into account.
4. High levels of heterogeneity were found across studies in all analyses. Estimates provided should, therefore, be interpreted with caution.
5. Studies included in the correlational analyses mostly utilized university samples, and many of the diagnostic studies used registry codes without detailing the interview protocols used. As a result, generalizability is limited.

## INTRODUCTION

Autism is a developmental condition characterized by difficulties in social communication and interaction, as well as restrictive and repetitive behaviours and interests (American Psychiatric Association, 2013). It is estimated that 1% of children internationally have a diagnosis of autism (Zeidan et al., 2022), though recent data from the Centers for Disease Control and Prevention (CDC) suggests this figure may be as high as 2.8% (CDC, 2023). Lower estimates are often found in non‐Western, particularly developing countries, likely due to limitations in diagnostic capacity, but cultural stigmas may also play a significant role (Issac et al., 2025; Samadi et al., 2012). While prevalence in recent years appears to be on the rise, this upward trend is likely the result of refinement in the diagnostic criteria and increased awareness (Talantseva et al., 2023). These refinements have included a movement away from strict autistic diagnostic categories, such as Autistic Disorder and Asperger's Disorder, towards a spectrum‐oriented view, acknowledging variability in symptom presentation and severity. Differences have been highlighted, however, in the diagnostic frameworks used to diagnose autism, with the ICD‐11 allowing for a broader range of symptom combinations than the DSM‐5, enabling a more diverse perspective of autism while also raising concerns about diagnostic specificity (Kamp‐Becker, 2024). Further, autistic traits, particularly social deficits, have been found to be common within general population samples, though scores largely fall under clinical cutoffs and are significantly lower than those with an autism diagnosis (Constantino & Todd, 2003; Ruzich et al., 2015).

Psychosis, on the other hand, describes a spectrum of unshared experiences, such as delusional thoughts and hallucinations (American Psychiatric Association, 2013). Meta‐analytic evidence suggests the international median lifetime prevalence of psychotic disorders to be approximately 0.749% (Moreno‐Küstner et al., 2018), though some population estimates have this as high as 3.5% (Perälä et al., 2007). Beyond formal diagnoses, much research has examined individuals at Clinical Risk of Psychosis (CHR‐P), a sub‐clinical state of attenuated psychotic symptomology, usually occurring in brief, intermittent windows. These individuals, while not yet meeting the criteria for any psychotic disorder, are at increased risk of doing so in the future, with 25% developing a psychotic disorder within 3 years (Salazar De Pablo, Radua, et al., 2021; Salazar De Pablo, Woods, et al., 2021). Within the general population, the prevalence of CHR‐P is estimated to be around 1.7%, while in clinical samples, this prevalence is as high as 19.2% (Salazar De Pablo, Radua, et al., 2021; Salazar De Pablo, Woods, et al., 2021). Further, data from the World Health Organization would suggest that 7.8% of individuals internationally have had psychotic experience, hallucinations being most prominent (McGrath et al., 2015).

Recent meta‐analyses have suggested that autistic individuals are at an increased risk of experiencing psychotic symptoms (Kiyono et al., 2020; Vaquerizo‐Serrano et al., 2022), as well as an increased risk of developing a psychotic disorder (Lai et al., 2019; Zheng et al., 2018). Further, those with psychosis have been found to have a greater prevalence of autistic traits and are more likely to be diagnosed as autistic (De Crescenzo et al., 2019; Kincaid et al., 2017) than those without. As for how the co‐occurrence of the two conditions manifests, Larson et al. (2017) found that autistic individuals with psychosis were more likely to be diagnosed with atypical psychosis than those with psychosis alone, with the duration of psychotic episodes generally not meeting the six‐month minimum required for a DSM‐IV‐TR diagnosis of schizophrenia. They were also less likely to have stereotyped interests and behaviours compared to autistic individuals without psychosis. Further evidence of how the comorbidity presents itself is, however, limited.

As for what causes this association, there are several candidate explanations. Biological explanations are often cited, with evidence to suggest that the two conditions are associated with a multitude of shared genetic and chromosomal abnormalities (Rapoport et al., 2009; Rees et al., 2021; Sebat et al., 2009). Further, there is evidence to suggest that both conditions share several prenatal and perinatal risk factors, including parental age, birth complications, season of birth and birth weight (Davies et al., 2020; Gardener et al., 2009, 2011). Traumatic experience may also partially explain the relationship, with a recent analysis of the Avon Longitudinal Study of Parents and Children cohort study finding that traumatic experience mediated the association between autistic and psychosis traits by 41% (Dardani et al., 2023).

Relatedly, diagnostic complications may also play a role in the association. It has been suggested that autistic children may have difficulty interpreting questions regarding psychosis due to their propensity to interpret language hyperliterally (Sullivan et al., 2013). Supporting this, Wilson (2018) found that adolescents who had difficulty interpreting non‐literal language, whether autistic or neurotypical, had greater difficulty interpreting the ambiguous language used in the items of the Structured Interview for Psychosis‐Risk Syndromes (SIPS) pertaining to positive symptoms. Though not a psychosis measurement, similar interpretative issues have been identified with the Suicide Behaviours Questionnaire‐Revised, with autistic adults reporting difficulty in understanding and responding to certain questions (Cassidy et al., 2020).

Furthermore, psychological factors may lead to diagnostic complications. Gesi et al. (2024) highlight that autistic individuals may present with ‘pseudo‐psychotic’ traits, spanning both positive and negative domains. For example, sensory hypersensitivities and echolalia, both common traits of autism, may be mistaken for hallucinations, while social communication issues may resemble negative psychotic symptoms. Further, special interests or atypical behaviours may appear as bizarre ideas or disorganized symptoms. Such overlaps also appear with attenuated psychotic symptoms, though findings from (Vaquerizo‐Serrano et al., 2022) suggest that autistic and non‐autistic individuals at CHR‐P have comparable rates of transition to psychosis, suggesting this association reflects not only trait overlap, but a genuine risk. Further, evidence from case series studies suggests that autistic individuals may receive a misdiagnosis of psychosis (Dossetor, 2007; Ying et al., 2023) due to clinician difficulty in differentiating between diagnostic criteria, such as negative symptoms and catatonic behaviour. Social difficulties or asociality, for example, may be both an autistic trait and a negative psychotic symptom, and atypical autistic behaviours (including stimming) may be seen as disorganized psychotic behaviour. As such, it is important that the methods used to assess this association are considered. A better understanding of the aetiology of the comorbidity and how it manifests will enable better clinician understanding of presentations and symptom combinations, as well as the opportunity for better measure development designed with autistic individuals in mind.

## OBJECTIVES

Our aim was, therefore, to conduct meta‐analyses on studies examining the association between autism and psychosis across both clinical and sub‐clinical trait measures and to examine whether the strength of this association is moderated by the measurement methods used. Three research questions were proposed:

- Research Question 1: Is there an association between autistic traits and psychosis‐spectrum traits in general population samples?
- Research Question 2: Are individuals with a diagnosis of one condition more likely to be diagnosed with the other compared to the general population?
- Research Question 3: Do individuals with a diagnosis of either condition show elevated traits associated with the other, compared to general population controls?

Three separate primary analyses were conducted. The first examined data from correlational studies that used trait/symptom score measures to assess both conditions within general population samples. A previous meta‐analysis conducted a similar analysis (Zhou et al., 2019), finding no moderating effect of measure used, so this first analysis aimed to update these findings. The second examined data from studies that compared comorbidity rates of the two conditions to general population prevalence data. Finally, the third examined whether the diagnosis of either condition was associated with an increased trait/symptom score of the other, compared to general population control samples.

## METHODS

### Design

This study followed the Preferred Reporting Items for Systematic Reviews and Meta‐analyses (PRISMA; Page et al., 2021) guidelines and was preregistered on PROSPERO (ID: 368498).

Research Question 1 examined studies reporting associations between autistic and psychosis‐spectrum trait scores. Psychosis‐spectrum traits were defined broadly to include both schizotypal characteristics, stable personality traits associated with psychosis risk and psychotic‐like experiences (PLEs), which are often transient positive psychotic phenomena. Although these are two different constructs of psychosis‐spectrum, both provide estimates for psychosis‐proneness within the general population (Debbane et al., 2015; Linscott & Van Os, 2013).

Research Question 2 examined studies that reported the prevalence of one condition in a sample with the other (or vice versa), relative to general population samples. Although most included studies examined schizophrenia, to allow for a broader estimate of co‐occurrence with autism, psychosis‐spectrum diagnoses and states, including schizoaffective disorder, first‐episode psychosis (FEP), non‐specified psychotic disorders and non‐clinical groups experiencing clinical high risk of psychosis, were combined as ‘psychosis‐spectrum’ disorders.

Finally, Research Question 3 examined whether individuals with a diagnosis of either autism or a psychotic‐spectrum disorder (as defined above) showed elevated scores on the other condition's trait measures. This analysis aimed to assess whether the presence of a clinical diagnosis corresponded to elevated traits in the opposing domain. These analyses utilized the same definitions for psychosis‐spectrum traits and conditions as RQ 1 and 2.

### Eligibility criteria

Studies were included if they met the following criteria:

1. Use a measure of autism or autistic traits
2. Use a measure of psychosis or psychosis symptoms
3. Provide data on comorbidity between autism and psychosis
4. Include human participants
5. Include a healthy control/general population control sample
6. Participants must not have other comorbid conditions
7. Mean age of participants is greater than or equal to 18 years of age
8. Reports quantitative data

### Information sources and search strategy

Studies were retrieved from systematic searches from PsycINFO (through EBSCO), MEDLINE (through EBSCO), CINAHL (through EBSCO), Embase (through Ovid) and Web of Science. A full breakdown of search terms and limiters used is reported in Appendix 1. Duplicates were identified and manually removed by use of the Systematic Review Accelerator De‐duplicator tool (Clark et al., 2020). The last systematic search of all listed databases was conducted on the fourteenth of November, 2024. Further studies were retrieved from previous similar meta‐analyses. A review of references of included studies was also conducted, but no new papers were retrieved.

### Selection process

An initial abstract screening was conducted by the first author and two voluntary research assistants. Each abstract was reviewed by the first author and at least one other reviewer. This process was conducted using RAYYAN (Ouzzani et al., 2016), a web‐based application for abstract screening. Accepted papers were then exported as a .csv file, and a full‐text screening was conducted. As before, each paper was reviewed by the first author and at least one other reviewer. At the end of both the abstract and full‐text screening, conflicting decisions were addressed with the addition of a third rater.

### Data collection process

Data was then extracted by the first author. Papers were reviewed for either measures of effect (i.e., Pearson's r, odds ratios) or data that could be used to compute measures of effect (i.e., mean trait scores to calculate standardized mean difference). Where possible, psychosis subscale data was collected to compute sub‐analyses. Autistic subscale data was not extracted due to limited reporting within included studies. Where papers did not report effect data, or data allowing for the computing of effects, corresponding authors were contacted. If authors did not respond to the initial request, a follow‐up email was sent after two working weeks. If authors did not respond to the data request after 3 months, papers were then excluded.

### Outcome measures

The primary outcome measure was the strength of effect between autism and psychosis, with an interest in if and how the various methods used to measure both conditions moderated the strength or directionality of the effect. These measures could have been categorical (i.e., Autism Diagnostic Interview‐Revised (ADI‐R) or Autism Diagnostic Observation Schedule (ADOS) for autism assessments) or continuous (i.e., Autism Quotient (AQ) for autism trait score). Participant age and gender data were also collected, if available, in order to conduct further moderation analyses.

### Study quality assessment and risk of bias assessment

Study quality and risk of bias was assessed by use of the AXIS Critical Appraisal of Cross‐Sectional Studies tool (Downes et al., 2016), designed for evaluating cross‐sectional studies. The tool consists of 20 items assessing the factors of study aims and design, sampling methods and sample characteristics, measurement validity, appropriateness of analyses and reporting transparency (see Table S1 in Supplement for the full list of items). Each item was primarily rated with either a ‘yes’ or ‘no’ response, though for instances in which criteria were partially met, or in which adherence was questionable, a score of ‘partial’ was given.

Risk of bias was assessed by reviewing responses to items regarding sampling methods, management of non‐responders and appropriateness of analyses. Based on these criteria, for each study, a qualitative judgement of ‘low’, ‘medium’ or ‘high’ risk of bias was made. Quality was assessed by reviewing responses to items pertaining to methodological strength, validity of measurements used, internal consistency of results and transparency of reporting of ethical approval and conflicting interests. Again, from these criteria, each study was assigned a qualitative judgement of ‘low’, ‘medium’ or ‘high’ quality.

### Effect measures

Four effect measures were used in analyses. For the first research question, both Pearson's *r* and Spearman's *rho* were used. As the effect range of both measures exists between −1 and 1, the effects were combined into a single analysis. For the second research question, log odds ratios were calculated and then converted back to odds ratios to maintain symmetry in analysis (Borenstein et al., 2009), and for the third research question, standardized mean difference was calculated.

### Synthesis methods

Analysis was conducted by use of R Studio, using the ‘metafor’ package. Intercept‐only random effects models were used to compute the main effect size of each analysis and meta‐regressions were conducted to examine the moderating effect of measure used, directionality (whether psychosis diagnosis was examined in a sample of autistic individuals or vice versa), age and gender. Only measures used three or more times were included in the moderation analyses with the aim of reducing outlier bias. The risk of publication bias was estimated by the use of Egger's tests. Both *I* ^2^ and Cochran's Q were calculated to measure the variation between studies accounted for by heterogeneity, and Baujat plots were used to identify the contribution to heterogeneity of each study. Forest plots and Baujat plots for each analysis were generated using the metafor package for R.

Some of the included studies did not use the aforementioned measures to calculate their effect. Some reported data tables, containing diagnostic prevalence data for examined groups. Where possible, this data was used to calculate ORs, using the metafor package in R. Other studies reported mean trait scores for clinical and general population samples, which were used to calculate SMD.

## RESULTS

### Research question 1: Correlational analyses

#### Study selection and characteristics

Thirty‐six papers reported correlational data for the first analysis. From these, 31 data points were extracted examining correlations between autistic traits score and overall psychosis‐spectrum trait score data, as well as 38 data points examining correlations between autistic traits and positive psychosis‐spectrum traits, 30 for negative and 23 for disorganized. The most common autism measurement used within these was the Autism Quotient (AQ; Baron‐Cohen et al., 2001), used in 30 of the included papers, and the most common psychosis‐spectrum trait measurement was the Schizotypal Personality Questionnaire (SPQ; Raine, 1991), used in 12. An overview of each of the included studies for these analyses can be found in Table 1.

**TABLE 1:** Study characteristics of correlational analyses.

Name, year	*N*	% of males	Mean age (range)	Sample type	Autism measure	Psychosis measure	Overall (*k* = 31)	Positive (*k* = 38)	Negative (*k* = 30)	Disorganized (*k* = 23)
Abu‐Akel, Apperly, Wood, Hansen, and Mevorach (2017)	202	21.29%	21.45 (NR)	University	AQ	CAPEp	NA	0.31	NA	NA
Abu‐Akel, Apperly, Wood, and Hansen (2017)	24	20.83%	21.21 (NR)	Community	AQ	CAPEp	NA	0.28	NA	NA
Abu‐Akel et al. (2018)	58	22.41%	20.95 (18–34)	Community	AQ	CAPEp	NA	0.442	NA	NA
Abu‐Akel et al. (2018)	69	27.54%	26.26 (17–36)	Community	AQ	CAPEp	NA	0.215	NA	NA
Blain et al. (2017)	107	29.91%	19.73 (NR)	University	AQ‐10	SPQ	0.4	0.28	0.36	0.36
Butler et al. (2015)	194	43.80%	21.31 (NR)	Community	AQ‐10	SPQ‐B	0.249	0.115	0.197	0.249
Choteau et al. (2016)	347	55.90%	21.8 (NR)	University	AQ	SPQ	0.55	0.294	0.605	0.368
Claridge and McDonald (2009)	77	42.86%	20.7 (17–27)	University	AQ	O‐LIFE: Introversive Anhedonia	NA	NA	0.5	NA
Bin Dawood et al. (2023)	71	35.21%	22.42 (18–37)	University	AQ	SPQ‐BR	0.455	NA	NA	NA
Del Giudice et al. (2010)	199	49.70%	22.6 (21–35)	Uni/Community	AQ	SPQ	NA	0.24	0.63	NA
Del Giudice et al. (2014)	151	50.30%	25.9 (18–38)	Uni/Community	AQ	SPQ	0.473	0.2	0.619	NA
Dinsdale et al. (2013)	605	37.19%	19.4 (NR)	University	AQ	SPQ‐BR	0.43	0.21	0.5	0.29
Ford et al. (2017)	835	25.30%	26.15 (18–40)	University	AQ	SPQ	0.679	0.494	0.733	0.614
Georgiou et al. (2021)	508	54.72%	26.51 (18–80)	Community	AQ	MSS‐B	0.341	0.418	0.224	0.21
Gillespie et al. (2017)	55	29.09%	20 (18–37)	University	AQ	CAPEp	NA	0.472	NA	NA
Gong et al. (2017)	2469	27.70%	18.75 (NR)	University	AQ	SPQ	0.419	0.213	0.515	0.353
Horder et al. (2014)	772	28.11%	NR (<17–>61)	University (Staff inc.)	AQ	CAPS	NA	0.333	NA	NA
Hurst et al. (2007)	607	22.24%	19.26 (17–55)	University	AQ	SPQ	0.47	0.25	0.53	0.32
Karvelis et al. (2018)	39	38.50%	23 (18–69)	Community	AQ	SPQ	0.602	NA	NA	NA
Karvelis et al. (2018)	83	49.40%	25.7 (18–69)	Community	AQ	RISC	NA	0.074	NA	NA
Louzolo et al. (2017)	925	100.00%	24.98 (18–35)	Community	AQ	PDI	NA	0.192	NA	NA
Mamah et al. (2022)	9564	52.92%	21.2 (15–25)	Community	AQ (Adolescent version)	pWERCAP	0.19	NA	NA	NA
Martinez et al. (2021)	7353	43.19%	51.2 (NA)	Community	AQ‐20	PSQ	0.05	NA	NA	NA
Mealey et al. (2014)	144	39.58%	25.3 (18–55)	Uni/Community	AQ	SPQ	0.54	0.41	0.6	0.36
Melchers et al. (2015)	107	93.00%	22.21 (NA)	University	AQ	CAPE	0.583	0.354	0.591	NA
Milne et al. (2017)	30	76.67%	35.37 (19–68)	Community	SRS	CAPS	0.16	NA	NA	NA
Nenadić et al. (2021)	264	21.59%	20.66 (NA)	University	AQ	SPQ‐B, O‐LIFE, MSS, CAPEp & CAPEn	0.53	0.35	0.47	0.44
Nenadić et al. (2021)	376	34.84%	24.04 (NA)	Community	AQ	SPQ‐B, O‐LIFE, MSS, CAPEp & CAPEn	0.5	0.25	0.45	0.38
Nenadić et al. (2021)	264	21.59%	20.66 (NA)	University	AQ	CAPEp & CAPEn	NA	0.384	0.516	NA
Nenadić et al. (2021)	376	34.84%	24.04 (NA)	Community	AQ	SPQ‐B	0.493	0.265	0.452	0.375
Nenadić et al. (2021)	376	34.84%	24.04 (NA)	Community	AQ	O‐LIFE	0.505	0.265	0.493	0.41
Nenadić et al. (2021)	264	21.59%	20.66 (NA)	University	AQ	O‐LIFE	0.54	0.326	0.476	0.473
Nenadić et al. (2021)	376	34.84%	24.04 (NA)	Community	AQ	MSS	0.497	0.229	0.405	0.357
Nenadić et al. (2021)	264	21.59%	20.66 (NA)	University	AQ	MSS	0.511	0.327	0.404	0.399
Raynal, Goutaudier, et al. (2016)	294	33.67%	20.31 (18–26)	University	AQ‐Short	SPQ‐B	0.414	0.142	0.448	0.303
Raynal, Melioli, et al. (2016)	466	33.70%	20.58 (18–24)	Uni/Community	AQ‐10	SPQ‐BR	0.317	0.226	0.233	0.272
Russell‐Smith et al. (2011)	362	24.03%	18.7 (NA)	University	AQ	O‐LIFE	0.46	0.15	0.51	0.38
Russell‐Smith et al. (2011)	639	30.67%	19.1 (NA)	University	AQ	O‐LIFE (MODIFIED)^a^	0.51	0.19	0.56	0.38
Russell‐Smith et al. (2013)	284	28.20%	20.1 (NA)	University	AQ: Social Skills subscale	O‐LIFE: Introversive Anhedonia	NA	NA	0.74	NA
Salminen et al. (2022)	296	35.81%	19.6 (NA)	University	AQ	SPQ‐B—Cognitive‐Perceptual	NA	0.174	NA	NA
Sampson et al. (2021)	653	17.30%	39.3 (18–65)	Community	AQ	CAPEp	NA	0.509	NA	NA
Shi et al. (2017)	864	NR	NA	University	AQ	SPQ	0.443	0.249	0.529	0.381
Sierro et al. (2016)	921	27.80%	22.2 (18–30)	University	AQ	sO‐LIFE	0.419	0.199	0.479	0.355
Wakabayashi et al. (2012)	662	49.55%	18.9 (18–27)	University	AQ (Japanese)	SPQ	0.483	0.1	0.44	0.24
Zhou et al. (2021)	115	40.00%	21.37 (18–30)	Community	AQ	SPQ	0.425	NA	NA	NA
Ziermans et al. (2021)	337	45.40%	38.5 (16–50)	Community	AQ	CAPE	NA	0.408	0.309	NA

Abbreviations: AQ, Autism Spectrum Quotient; CAPE, Community Assessment of Psychic Experience; CAPEn, Community Assessment of Psychic Experience—Negative subscale; CAPEp, Community Assessment of Psychic Experience—positive subscale; CAPS, Cardiff Anomalous Perception Scale; MSS(‐B), Multidimensional Schizotypy Scales (‐Brief); O‐LIFE, Oxford‐Liverpool Inventory of Feelings and Experiences; PDI, Peters' Delusion Inventory; PSQ, Psychosis Screening Questionnaire; pWERCAP, Washington Early Recognition Center Affectivity and Psychosis Screen; RISC, Rust Inventory of Schizotypal Cognitions; sO‐LIFE, Short Oxford‐Liverpool Inventory of Feelings and Experiences; SPQ—BR, Schizotypal Personality Questionnaire—Brief Revised; SPQ, Schizotypal Personality Questionnaire; SPQ‐B, Schizotypal Personality Questionnaire‐Brief; SRS, Social Responsiveness Scale.

^a^ Modified O‐LIFE—Addition impulsive nonconformity subscale of 10 items.

One included study for these analyses measured the association using several questionnaires, across two samples. For the overall analyses, we combined these correlations into a single effect. For the moderation analyses, however, we included two data points from the same sample, as bias testing concluded no significant differences in effect whether the study was excluded or not.

**FIGURE 1:**
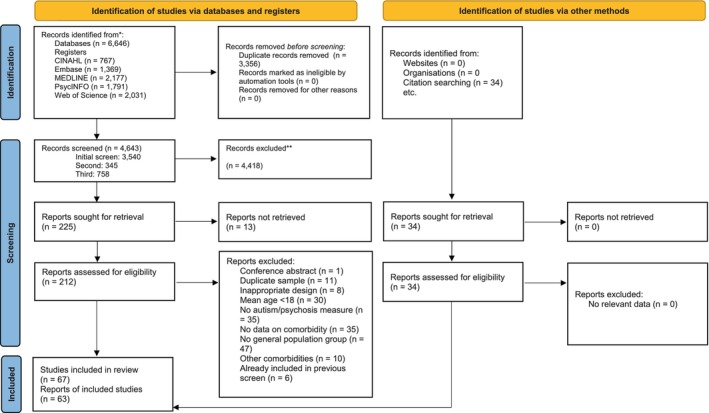
PRISMA flow diagram of systematic review process.

Figure 1 presents a PRISMA (Preferred Reporting Items for Systematic Reviews and Meta‐Analyses; Page et al., 2021) flowchart of the selection process for all included papers.

#### Risk of bias and quality assessment

Of the 36 papers, only seven were deemed to have a low risk of bias, while the remaining 29 were deemed to have a medium risk. This risk was most commonly introduced due to issues relating to sampling representativeness and lack of reporting of, or adjustment to, non‐response bias. As for study quality, only eight studies were deemed to be of high quality, while the remaining 31 were deemed to be of medium quality. This was primarily due to few studies making attempts to justify sample sizes, which were often small convenience samples. None of the included studies contained alarming risk of bias concerns, and all were generally methodologically robust.

#### Results of synthesis

**FIGURE 2:**
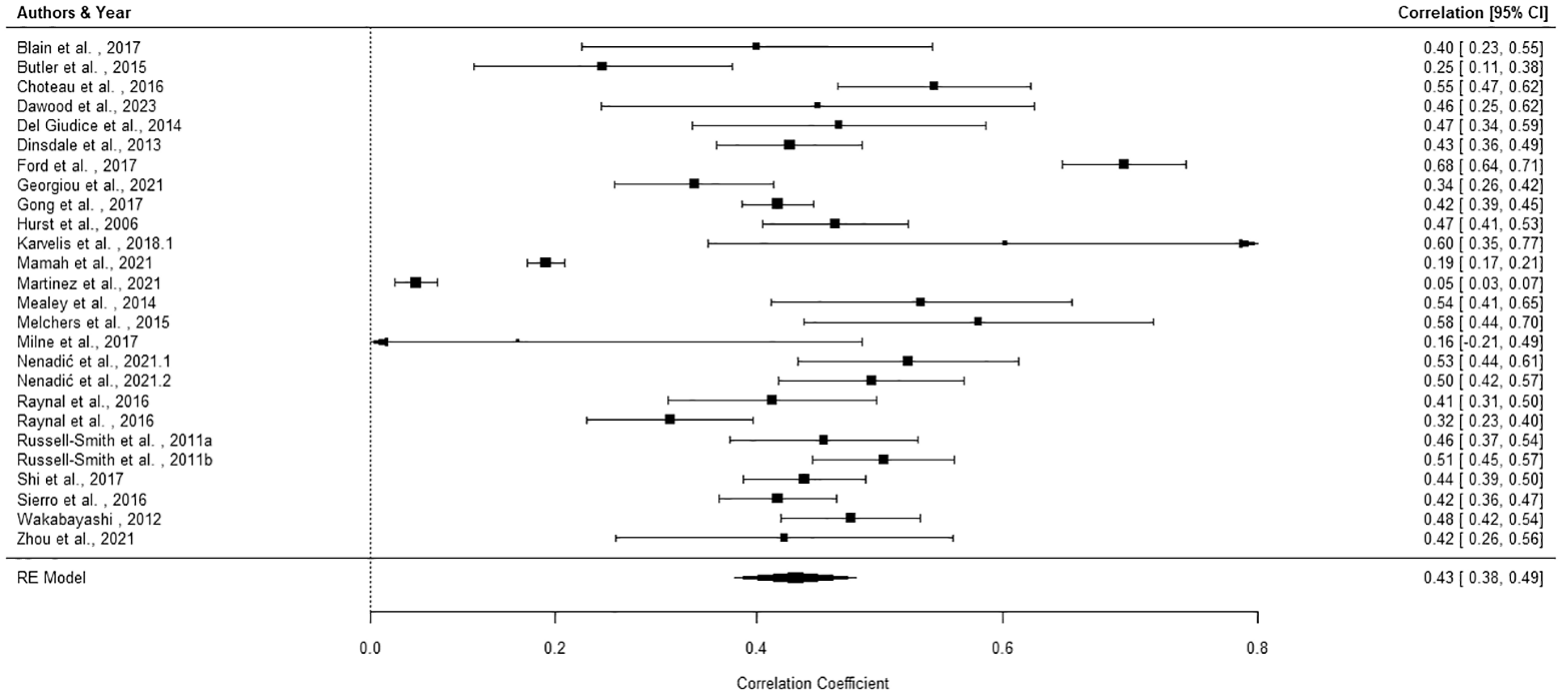
Forest plot of studies examining the association between autistic and psychotic symptoms.

The intercept‐only model for the analysis of correlation coefficients revealed a significant positive effect between autism trait and overall psychosis‐spectrum trait scores (*r* = .435, 95% CI = 0.379–0.487, *p* < .0001, *k* = 26). A forest plot of the effect sizes of each study, as well as the overall effect size, is presented in Figure 2. Heterogeneity between the studies was considerable (*I* ^2^ = 95.79%; *Q* (df = 25) = 1154.0492, *p* < .0001). Increased mean age of samples was significantly associated with decreased effect sizes (*p* = .008), but no moderating effect of proportion of males was found. Measurement type significantly moderated the association. A moderate association was found between autistic and overall schizotypal trait scores (*r* = .457, 95% CI = [.41–.51], *p* < .0001, *k* = 20), while no significant association was found with PLE measures (*r* = .259, 95% CI [−.009, .492], *p* = .058, *k* = 4). The Egger's test found no significant funnel plot asymmetry, suggesting that there is no significant risk of publication bias. A Baujat plot revealed that three papers had significant influence on the heterogeneity (see Figure S4 in Supplement).

Sub‐analyses found a large effect between overall autistic traits and negative psychosis‐spectrum traits (*r* = .503, 95% CI = 0.442–0.560, *p* < .0001, *k* = 24), and lower, yet still significant effects, were found for both positive (*r* = .274, 95% CI = 0.231–0.316, *p* < .0001, *k* = 32) and disorganized psychosis‐spectrum traits (*r* = .352, 95% CI = 0.304–0.399, *p* < .0001, *k* = 18) (see Figures S1–S3 in Supplement for forest plots for each of these analyses). Measurement type, again, significantly moderated the association. A small effect was found between autistic and positive schizotypal trait scores (*r* = .274, 95% CI = [.231, .316], *p* < .0001, *k* = 32), while no significant association was found with PLE measures (*r* = .259, 95% CI [−.009, .492], *p* = .058, *k* = 4). Egger's tests for each of these analyses found no significant funnel plot asymmetry. Heterogeneity for the positive (*I* ^2^ = 85.33%; *Q* (df = 31) = 222.2805, *p* < .0001), negative (*I* ^2^ = 94.22%; *Q* (df = 23) = 348.5369, *p* < .0001) and disorganized (*I* ^2^ = 86.34%; *Q* (df = 17) = 137.5886, *p* < .0001) analyses were again severe. Baujat plots revealed a number of problematic studies for each analysis (see Figures S5–S7 in Supplement). Problematic studies for each analysis generally produced significantly higher or lower effects. For the overall trait score analysis, most of the problematic studies used novel psychosis‐spectrum measures, but no other methodological differences were identified.

#### Research question 2—Diagnostic comorbidity

##### Study selection and characteristics

Seventeen papers had data on diagnostic comorbidity for the second analysis. An overview of included studies for these analyses can be found in Table 2. Thirteen of these papers examined the prevalence of psychosis‐spectrum disorders within autistic samples. Very few papers reported interview protocols of measurement tools for diagnosis, instead only reporting the diagnostic code they fell under.

**TABLE 2:** Study characteristics of odds ratio analysis.

Name, year	n1—Diagnosis group	Diagnosis sample type	Original diagnosis (criteria)	Comorbidity	n2—general population	% of males n1	% of males n2	n1 age (mean)	n2 age (mean)	Autism measure (classification)	Psychosis measure (classification)	OR (*k* = 16)
Amir et al. (2023)	737	Community	CHR‐P	Autism	275	57.53%	50.18%	18.49 (12–35)	19.76 (12–34)	SCID (DSM‐IV/5)	SIPS, SOPS (NA)	1.218
Chen et al. (2015)^a^	725	Clinical	Autism	Schizophrenia	27,540	77.24%	NR	18.34 (NA)	14.86 (NA)	DB (ICD‐9)	DB (ICD‐9)	203.632
Davignon et al. (2018)^a^	4123	Community	Autism	Psychoses	20,615	80.67%	80.67%	18.39 (14–25)	18.44 (14–25)	DB (ICD‐9)	DB (ICD‐9)	4.607
Fendrich et al. (2022)	40	Clinical	Psychosis	Autism	24	50.00%	45.83%	38.1 (18–55)	33.4 (18–55)	Self‐report	DIGS	0.605
Hand et al. (2020)^a^	4685	Community	Autism	Schizophrenia + psychotic disorders	46,850	67.77%	67.77%	NR (65+)	NR (65+)	DB (ICD‐10)	DB (ICD‐10)	26.84
Houghton et al. (2018)^a^	10,856	Community	Autism	Schizophrenia	21,712	80.69%	80.69%	18.76 (3+)	18.76 (3+)	DB (READ)	DB (Read)	6.736
Kendler et al. (2024)	2292	Community	Schizotypal Personality Disorder	Autism	11,460	48.5%	NR	NR	NR	DB (ICD‐9/10)	DB (ICD‐10)	19.76
Kohane et al. (2012)^a^	5276	Clinical	Autism	Schizophrenia	1,142,008	78.71%	NR	NR	NR	DB(ICD‐9)	DB (ICD‐9)	11.559
Krieger et al. (2021)	24,667	Clinical	Schizophrenia	Autism	24,667	63.08%	63.08%	NR (18–70)	NR (18–70)	DB (ICD‐9/10)	DB (ICD‐9)	7.01
Roy et al. (2015)^a^	50	Clinical	Asperger's Syndrome	Schizophrenia + psychotic disorders	4181	68.00%	50.30%	36.46 (20–62)	NR (18–65)	DB (DSM‐IV)	DB (DSM‐IV (German))	0.777
Schendel et al. (2016)^a^	20,492	Community	Autism	Schizophrenia, schizotypal or delusional disorders	1,892,412	77.60%	51.00%	NR (1.5–33)	NR (1.5–33)	DB (ICD‐8/10)	DB (ICD‐8/ICD‐10)	8.793
Solberg et al. (2019)^a^	7528	Community	Autism	Schizophrenia	1,653,575	72.10%	51.00%	26.2 (NR)	33.2 (NR)	DB (ICD‐10)	DB (ICD‐10)	11.122
Suen et al. (2024)^a^	291	Community	Autism	Psychotic disorder	1605	51.50%	39.80%	19.69 (15–24)	19.88 (15–25)	Self‐report	Self‐report	0.688
Supekar et al. (2017)^a^	4790	Community	Autism	Schizophrenia	1,842,575	NR	NR	NR (18+)	NR (18+)	DB (ICD‐9)	DB (ICD‐9)	4.476
Tint et al. (2023)^a^	10,646	Community	Autism	Psychotic disorders	42,607	69.75%	69.68%	NR (19–65)	NR (19–65)	DB (ICD‐9/10/DSM‐IV)	DB (ICD‐9/10/DSM‐IV)	13.04
Underwood et al. (2022)	7943	Community	Autism	Schizophrenia	25,941	75.49%	74.62%	NR (18+)	NR (18+)	(ICD‐10/READ v2)	(ICD‐10/Read v2)	7.763
Vohra et al. (2017)^a^	1772	Community	Autism	Schizophrenia	5320	71.40%	71.50%	NR (22–64)	NR (22–64)	DB (ICD‐9‐CM)	DB (ICD‐9)	1.595

Abbreviations: DB, Database cohort (no measurement information reported); DIGS, Diagnosis Interview for Genetic Studies; DSM, Diagnostic and Statistical Manual of Mental Disorders; ICD, International Classification of Diseases; SIPS, Structured Interview for Psychosis‐Risk Syndromes; SOPS, Scale for Assessment of Psychosis‐Risk Syndromes.

^a^ Marked studies examined psychosis diagnosis within autistic populations; unmarked studies examined autism diagnosis within samples with psychosis.

##### Risk of bias and quality assessment

Of the 17 papers, 13 demonstrated low risk of bias, often utilizing large, representative population datasets. Issues regarding non‐response bias were, however, present in four papers, resulting in medium risk of bias ratings. Quality was also generally high, with the same 13 papers being deemed high quality. The remaining four papers, however, were rated medium quality, due to the lack of justification in sample size, as well as a lack of clarity in reporting of measurements used.

##### Results of synthesis

**FIGURE 3:**
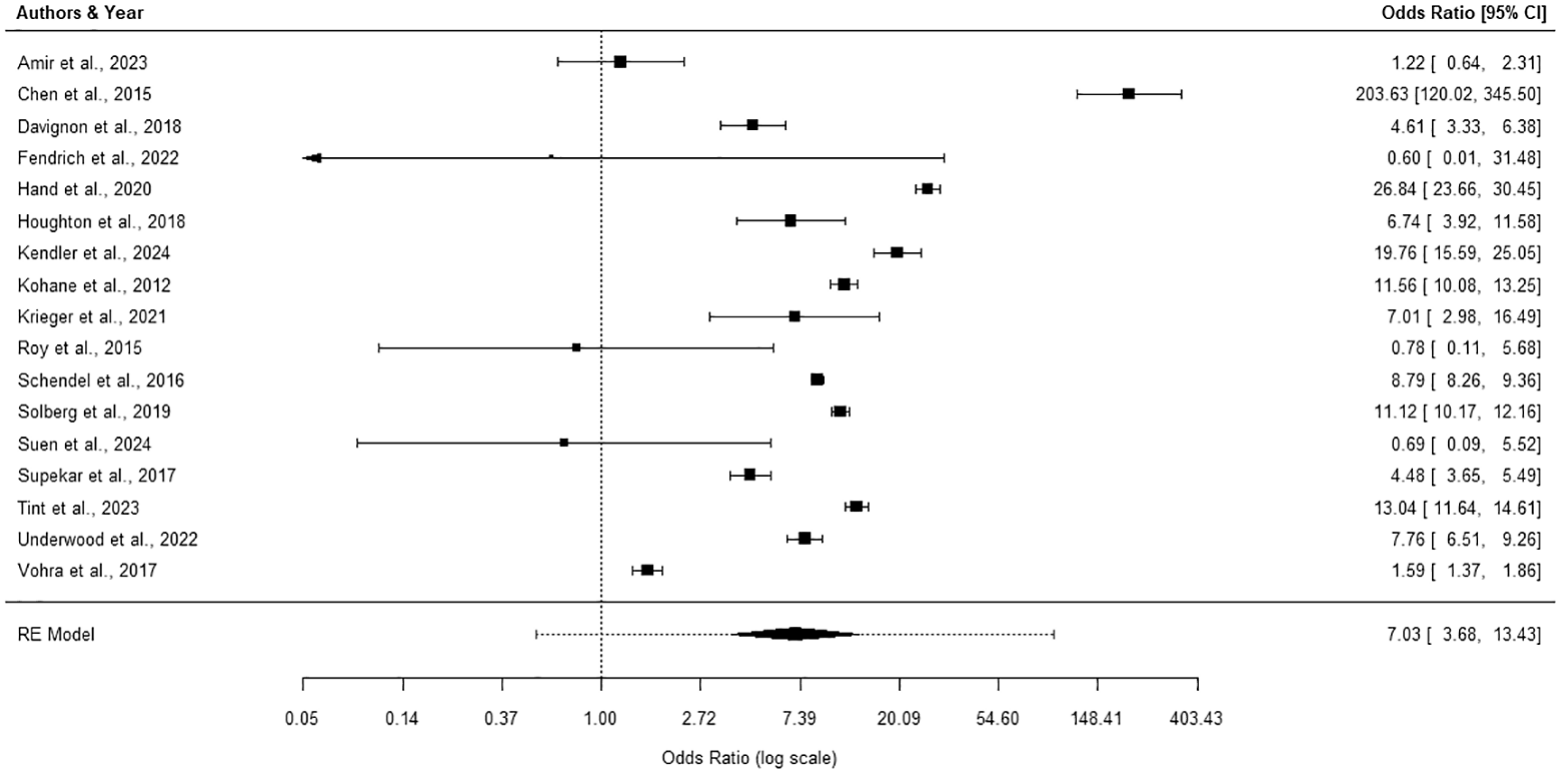
Forest plot of studies included in odds ratio analyses.

The intercept‐only model for the analysis of odds ratios, again, revealed a significant positive effect (OR = 7.03, 95% CI = 3.68–13.43, *p* < .001, *k* = 17), suggesting that individuals with one condition were over seven times more likely to be diagnosed with the other compared to the general population. A forest plot of the effect sizes of each study, as well as the overall effect size, is presented in Figure 3. Due to limited data, we were not able to examine the moderating effect of the measure used. No moderating effects of age, gender or study directionality were found. Heterogeneity between the studies was considerable (*I* ^2^ = 99.57%; *Q* (df = 16) = 1145.6134, *p* < .0001). A Baujat plot revealed that the high heterogeneity was largely the result of a single paper (Chen et al., 2015), likely due to its exceptionally large effect (see Figure S8 in the Supplement). Removing this paper, however, did not significantly reduce overall heterogeneity, suggesting that heterogeneity was widespread across included studies. Egger's test did find significant funnel plot asymmetry, highlighting a risk of publication bias.

#### Research question 3—Effect of diagnosis on trait score

##### Study selection and characteristics

Thirteen papers reported mean trait score data of both a clinical sample (autism or psychosis‐spectrum) and a general population sample. An overview of included studies for these analyses can be found in Table 3.

**TABLE 3:** Study characteristics of standardized mean difference analysis.

Name, year	n1—Diag. group	Diag. Sample type	Diagnosis	n2—Gen. population	% of males n1	% of males n2	n1 mean age (range)	n2 mean age (range)	Autism measure (classification)	Psychosis measure (classification)	SMD (*k* = 13)
Craig et al. (2004)^a^	17	Clinical	Asperger's syndrome	16	88.24%	68.75%	24.12 (NR)	29.44 (NR)	NR (ICD‐10)	Paranoia Scale	1.406
de Bildt et al. (2016)	18	Clinical	Schizophrenia	21	100.00%	100.00%	37 (19–61)	34.24 (21–53)	ADOS	Clinician (not specified)	1.386
Lugnegård et al. (2015)	36	Clinical	Schizophrenic psychosis	49	63.89%	38.78%	29.1 (NR)	28.6 (NR)	AQ	SCID‐I (DSM‐IV)	1.459
Martinez et al. (2017)	36	Clinical	Schizophrenia	20	83.33%	85.00%	23.4 (NR)	23.4 (NR)	AQ	DIGS (DSM‐IV‐TR)	0.788
Matsuo et al. (2015)	44	Clinical	Schizophrenia	65	45.45%	27.69%	36.9 (25–59)	42.2 (25–59)	SRS‐A	MINI/PANSS (DSM‐IV‐TR)	1.242
Milne et al. (2017)^a^	30	Clinical	Autism	30	76.67%	63.33%	32.65 (19–68)	35.37 (19–70)	NR	CAPS	1.744
Pinkham et al. (2008)^a^	12	Clinical	Autism	12	100.00%	100.00%	24.08 (18–35)	27.08 (18–35)	NR (DSM‐IV)	Paranoia Scale	1.292
Sasamoto et al. (2011)	20	Clinical	Schizophrenia	25	70.00%	64.00%	34.5 (NR)	34.5 (NR)	AQ	SCID (DSM‐IV)	1.578
Suen et al. (2024)^a^	486	Community	Probable autism	1295	NR	NR	NR (NR)	NR (NR)	AQ‐10	PQ‐B	−0.208
Upthegrove et al. (2018)	99	Clinical	FEP	381	67.68%	21.00%	25.61 (16–35)	20.61 (17–39)	AQ	NR (ICD‐10)	0.845
Wouters and Spek (2011)	21	Clinical	Schizophrenia	21	100.00%	100.00%	40.9 (18–65)	40.8 (18–65)	AQ	SCID‐I (DSM‐IV‐TR)	1.498
Zhang et al. (2016)	37	Clinical	Schizophrenia	38	81.08%	78.95%	20.95 (NR)	21.32 (NR)	AQ (Chinese)	NR (DSM‐IV‐TR)	2.176
Ziermans et al. (2021)	504	Clinical	Psychotic disorder	337	72.42%	45.40%	33.4 (16–50)	38.5 (16–50)	AQ	NR (DSM‐IV‐TR)	1.153

Abbreviations: ADOS, Autism Diagnostic Observation Schedule; AQ, Autism Spectrum Quotient; CAPS, Cardiff Anomalous Perception Scale; DIGS, Diagnosis Interview for Genetic Studies 3.0; DSM, Diagnostic and Statistical Manual of Mental Disorders; MINI, Mini‐International Neuropsychiatric Interview; PANSS, Positive and Negative Symptoms Scale; PQ‐B, Prodromal Questionnaire‐Brief; SCID, Structured Clinical Interview for DSM‐IV; SRS‐A, Social Responsiveness Scale for Adults.

^a^ Marked studies examined psychosis‐spectrum traits within autistic populations; unmarked studies examined autistic traits within samples with psychosis.

##### Risk of bias and quality assessment

Of the 13 studies, only two were deemed to have a low risk of bias. The remaining 11 were deemed to have a medium risk of bias, primarily due to issues in sample representativeness, as well as limited reporting of possible non‐response bias. Regarding quality, the same two studies were marked as being of high quality, while the remaining 11 were deemed to be of medium quality. This was, again, largely due to a lack of justification of sample size.

#### Synthesis of results

**FIGURE 4:**
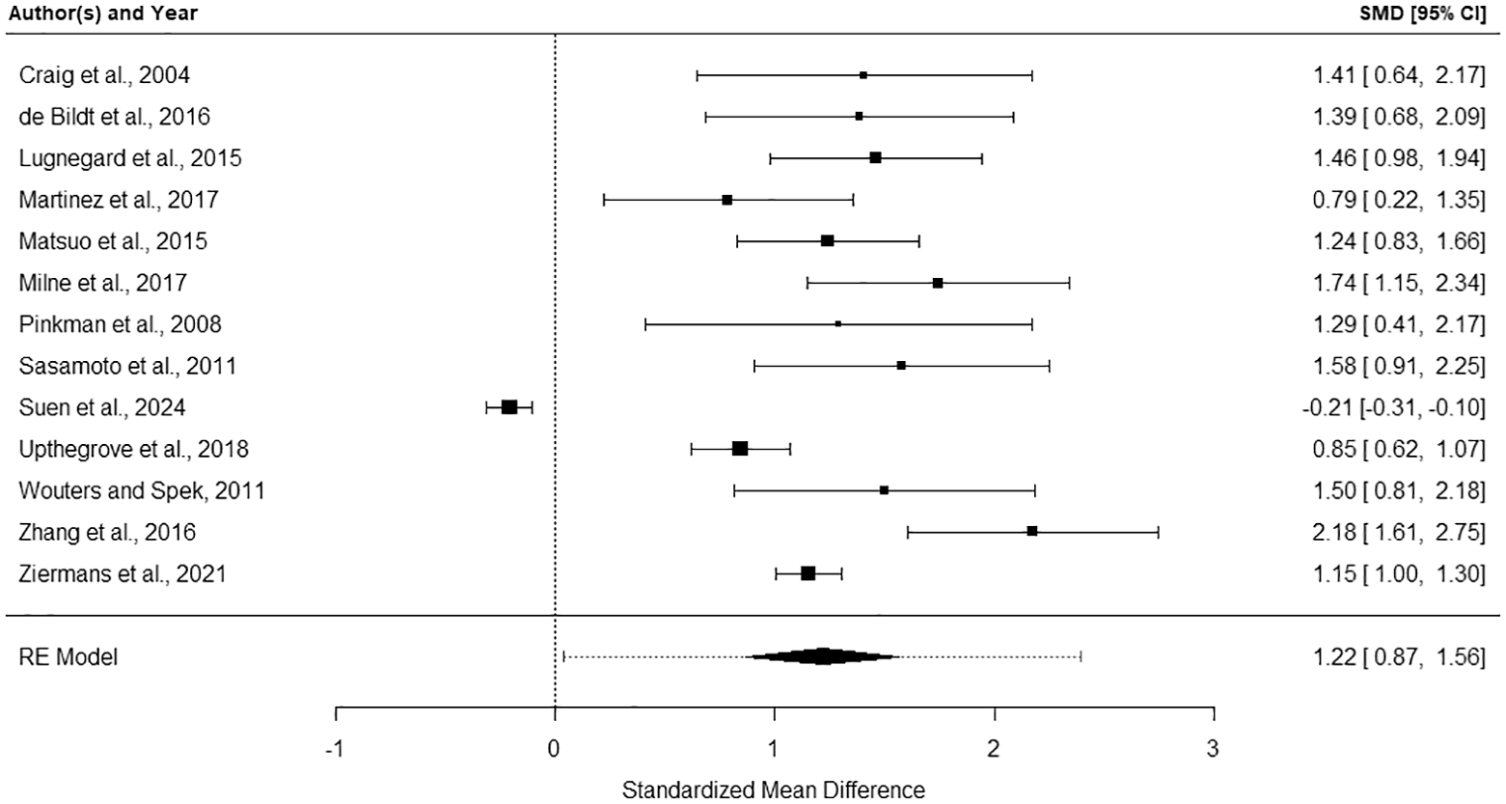
Forest plot of studies included in the standardized mean difference analyses.

The intercept‐only model for the analysis of standard mean differences also revealed a significant positive effect, with diagnosis of one condition predicting an increased trait score of the other (*d* = 1.216, 95% CI = 0.87–1.563, *p* < .0001, *k* = 13). A forest plot of the effect size of each study, as well as the overall effect, is presented in Figure 4. As with the previous analysis, due to limited data, we could not examine the moderating effect of measure used. No moderating effects were found for directionality, mean age or proportion of males. Heterogeneity between studies was considerable (*I* ^2^ = 92.96%; *Q* (df = 12) = 370.3137, *p* = 0.001). A Baujat plot revealed that heterogeneity was largely explained by a single paper (Suen et al., 2024) (see Figure S9 in Supplement). If this paper was removed, heterogeneity dropped significantly (*I* ^2^ = 68.02%; *Q* (df = 11) = 29.7086, *p* = 0.0018). Egger's test identified significant funnel plot asymmetry, suggesting a risk of publication bias. If, however, the abovementioned paper was removed from analyses, Egger's test became insignificant.

## DISCUSSION

These meta‐analyses provide an updated review of the association between autism and the psychosis spectrum. In line with previous research, a significant association was found between the two conditions, regardless of the methods and measures of assessment used. In line with the findings from Zhou et al. (2019) (*k* = 19), Research Question 1 revealed that within the general population samples, overall autism trait scores were significantly positively associated with overall psychosis‐spectrum symptom scores. Significantly lower overlaps, however, were found with questionnaires examining PLEs (e.g., CAPE) and positive schizotypy (e.g., SPQ). Further in line with their findings, sub‐analyses revealed that negative psychosis symptoms were more strongly associated with autistic traits than positive or disorganized symptoms.

Research Question 2 revealed that individuals with a diagnosis of either autism or a psychosis‐spectrum condition were more likely to be diagnosed with the other, compared to general population samples. This finding is, again, supported by previous meta‐analyses (Lugo Marín et al., 2018; Varcin et al., 2022). Finally, Research Question 3 found that individuals with a diagnosis of either autism or a psychosis‐spectrum condition were more likely to score higher on trait score measures of the other condition, compared to general population samples. This finding is also in line with those of previous meta‐analyses (De Crescenzo et al., 2019; Kiyono et al., 2020; Vaquerizo‐Serrano et al., 2022). As for moderating variables, higher mean age was associated with smaller effects for the correlational analysis (RQ1), and the effect size for the association between a diagnosis of autism and psychosis (RQ2) was larger for males than females. For RQ2 and RQ3, it was not possible to examine the type of measure as a moderator due to a lack of sufficient data.

Given the results of our meta‐analyses, three key points need to be discussed: (i) the overlap between autistic traits and negative symptoms, (ii) diagnostic complexity and (iii) the relative lack of other moderation effects given the high heterogeneity.

- Although autistic traits were found to positively correlate with all three subdimensions of psychosis‐spectrum traits, the association was strongest with negative traits, suggesting considerable overlap with these constructs. This notion is supported by several studies examining overlap between autistic trait and psychosis‐spectrum symptom score measures. Regarding the Autism Quotient (AQ) and the Schizotypal Personality Questionnaire (SPQ), for example, the association between the ‘social skills’ and ‘communication’ subscales of the AQ and the ‘interpersonal’ subscale of the SPQ appears to explain a large degree of the association between overall score correlation (Ford et al., 2017; Hurst et al., 2007). Further, some have even found negative correlations between AQ scores and the Cognitive‐Perceptual (positive symptom) domain of the SPQ (Wakabayashi et al., 2012; Zhou et al., 2019). Of the included studies for our correlational analyses, 35% used a combination of these measures; 60% if including modified versions. As such, it is possible that the overall effects are partly explained by the similarity between these two questionnaires. Similar overlaps have also been identified between the AQ and the Brief version of the SPQ (SPQ‐B; Dinsdale et al., 2013), the Multidimensional Schizotypy Scale (MSS) and the Oxford‐Liverpool Inventory of Feelings and Experiences (Nenadić et al., 2021; Russell‐Smith et al., 2011).

This overlap is likely explained by a combination of factors. Conceptually, it may be the case that these traits are genuinely shared between autistic and psychotic conditions, leading to legitimate overlap in measurement. It may also be the case, however, that methodological blurring may be inflating the association, with certain items failing to adequately distinguish autistic and psychosis‐spectrum traits. Such blurring may also be present between items pertaining to autistic idiosyncrasies and disorganized psychosis traits (i.e., self‐perceptions of odd behaviours and speech). Finally, it could be that some features are transdiagnostic, possibly related to shared biological (e.g., genetic and chromosomal mutations) (Rapoport et al., 2009; Rees et al., 2021; Sebat et al., 2009) and environmental (e.g., pre‐ and perinatal) risk factors previously discussed (Davies et al., 2020; Gardener et al., 2009, 2011). These findings, coupled with both the modest effects of our positive symptoms analyses and the risk of item misinterpretation, call into question whether much of this research is truly measuring the association between autism and psychosis.

Attempts have been made, however, to mitigate the effect of this trait similarity. The Psychopathology in Autism Checklist (PAC), developed by Helverschou et al. (2009), is a 30‐item carer‐reported questionnaire examining symptoms of anxiety, depression, obsessive‐compulsive disorder and psychosis that do not overlap with symptoms of autism. To date, however, this questionnaire has not been widely used. More recently, Parvaiz et al. (2023) developed the schiZotypy Autism Questionnaire (ZAQ), a 134‐item self‐report questionnaire which attempts to better discriminate between symptoms of autism and schizotypy. The questionnaire, co‐designed with autistic individuals and individuals with schizophrenia, has not yet been validated, but once trialled, may well produce lower effects than studies using standard measures of psychosis symptomology, such as the SPQ. However, none of the included studies used these measures, so an evaluation of their association with autistic traits is outstanding.

ii As mentioned, it is possible that this comorbidity is partially explained by clinician difficulty in differentiating between displayed autistic and psychotic behaviours. Starling and Dossetor (2009) suggested some of the difficulties clinicians may face distinguishing between symptoms of the two conditions. They highlight that thought disorders and bizarre behaviours are symptoms of both autism and psychosis, that autistic idiosyncrasies and unusual world views may be misinterpreted as psychotic delusions and that self‐talk may be viewed as evidence of auditory hallucinations. While little research has yet examined the appropriateness of using existing clinical measures of psychosis within autistic populations, some research has examined the appropriateness of the SIPS (Wilson et al., 2020). Prior findings from these researchers found that clinicians, experienced in working with autistic individuals, deemed over 60% of its items as being problematic for use within autistic populations (Fleischman et al., 2016), for reasons of symptom overlap, poor wording and complexity. Efforts have been made, however, to improve the discriminative ability of assessment tools. In a sample of 50 autistic individuals and 40 individuals with schizophrenia, Nakamura et al. (2024) found that standard ADOS‐2 algorithms did not adequately differentiate between the two conditions, resulting in high rates of false positives. They found, however, that a predictive model using a subset of six ADOS‐2 items significantly improved discrimination. Further investigation into the discriminative validity of such assessment tools is essential to reduce the risk of false‐positive diagnoses and to ensure and expedite appropriate treatment.
iii For each of the analyses, particularly for the correlational and odds ratio, heterogeneity between studies was severe, though clear reasons for this were not identified. While we found some evidence of age and sex effects on the results of the correlation and odds ratio analyses, respectively, the remainder of our moderation analyses yielded insignificant results. The heterogeneity is, therefore, largely unexplained by the moderating factors we examined. Included papers, however, differed in design (e.g., sample characteristics), in measurement (structured interviews, registry data, self‐report measures) and in the examined constructs (traits, symptoms or disorders). Such methodological differences are likely to contribute to the observed variance, even if not detectable through formal analyses. It is also possible that there exist other unaccounted‐for factors that help to explain the association (i.e., the presence of other comorbid conditions, IQ or socio‐economic status).

### Strengths and limitations

This meta‐analysis provides an updated overview of the association between autism and psychosis, including more data points than previous analyses, and examines the association through a combination of the various methodologies by which it is measured, thereby providing a holistic overview of the comorbidity profile. It also provides a thorough attempt at examining the moderating effect of measures used, highlighting gaps in the literature. Despite this, there are, however, several limitations worth noting. Firstly, in our analyses, we excluded study samples if participants had other comorbid conditions. While doing so allowed for a clearer examination of the association between the two conditions, it leaves to question the possible moderating role of additional comorbid conditions. For example, research has found increased prevalence of attention deficit hyperactivity disorder (Stralin & Hetta, 2021) and obsessive‐compulsive disorder (Mawn et al., 2020) in samples with psychosis. It could, therefore, be the case that additional comorbidities, or particular combinations, better explain or further increase the risk of the comorbidity.

Secondly, in our review, we excluded grey literature, only including peer‐reviewed papers. While beneficial for ensuring the quality of included research, excluding grey literature may leave meta‐analyses vulnerable to publication bias (Nair & Borkar, 2023), overrepresenting studies with significant results (Conn et al., 2003; McAuley et al., 2000). The addition of grey literature would have been of particular interest in the OR and SMD analyses, considering significant risk of publication bias was identified for both.

There are also two limitations from the reviewed studies worth reporting. The first is the omission of key data. Most importantly, some of the studies identified in the review did not report effect data, or data required to calculate effects. Upon contacting the authors of these papers, few responded, and fewer still had access to the analysed data. Relatedly, for the OR and SMD analyses, while most studies reported by which guidelines diagnosis of either autism or psychosis was met (i.e., ICD or DSM), few reported the actual methods or interview protocols used to acquire diagnosis. For these reasons, we were unable to examine the moderating effect of measure for these analyses. It would, therefore, be of benefit, particularly for future research syntheses in this field, for papers to ensure such information is reported within their papers. To ensure data accessibility for meta‐analyses, open science practices including the use of data repositories are encouraged.

The second issue relates to the generalizability and appropriateness of the included samples. For the correlational analysis, in particular, many of the included studies used undergraduate samples. As a result, samples mostly consisted of young adults in their early twenties, with a disproportionately low number of male participants (38%). Sex differences have been observed in the onset of psychosis, generally occurring in males in their early twenties and in women in their late twenties (Li et al., 2016). Further, there is evidence of a second risk period for women, generally occurring around the mid‐forties (Grigoriadis & Seeman, 2002; Kirkbride et al., 2012). The use of such samples may, therefore, not be ideal in estimating general psychosis prevalence. Mean ages were more diverse within the OR and SMD analyses, though within these samples, there was instead an increased prevalence of males (70% and 79%, respectively). For these reasons, it is important for future research to also report data from the sexes individually.

## CONCLUSION

This review highlights a clear association between autism and psychosis, at both a trait and diagnostic level. Findings suggest, however, that at the trait level, part of this association may be explained by overlaps in expression and measurement, as opposed to pathological overlap. As such, the appropriateness of self‐report psychosis‐spectrum screening tools within autistic samples should be interpreted cautiously. For diagnostic purposes, structured clinical interviews should be conducted with explicit consideration of autism, ideally by clinicians with experience in both conditions. Gold‐standard interviews, such as the SCID, requiring interviewees to provide concrete examples of psychotic experiences may improve diagnostic accuracy, given that questions are worded carefully and that answers are interpreted with caution. Careful scrutiny of developmental history is essential, and collateral information from family, as well as behavioural observation, can provide further important context to better differentiate between innate autistic characteristics from the onset of psychotic symptoms.

These findings highlight several important directions for future research. Firstly, further understanding of how autistic individuals interpret psychosis‐related questions is required. Inquiry should be focused on commonly used screening tools (e.g., SPQ, CAPE) and interview protocols (e.g., SIPS), and if it is the case that interpretative issues are present, amendments or alternative methods of assessment must be established. Secondly, little is currently known regarding the aetiology of the comorbidity, and as such, further research should examine factors that may moderate the association. Thirdly, more research is needed examining how psychosis manifests within autistic individuals. There is some evidence to suggest that autistic individuals may be more likely to be diagnosed with an atypical type of psychosis, and less likely to be diagnosed with schizophrenia, compared to non‐autistic individuals (Larson et al., 2017), but more research of this kind is still required. With greater understanding of how the comorbidity manifests, clinicians will be able to better identify the onset of psychosis within autistic populations, facilitating earlier intervention and treatment.

## AUTHOR CONTRIBUTIONS

**Michael R. Miles:** Conceptualization; investigation; writing – original draft; methodology; funding acquisition. **Dennis Golm:** Conceptualization; supervision; methodology; writing – review and editing; funding acquisition. **Emma Palmer‐Cooper:** Conceptualization; supervision; methodology; writing – review and editing; funding acquisition.

## CONFLICT OF INTEREST STATEMENT

No conflicts of interest to declare.

## FUNDING INFORMATION

This research received no specific grant from any funding agency, commercial or not‐for‐profit sectors.

## Supporting information


Data S1.

## Data Availability

The data that support the findings of this study are available from the corresponding author upon reasonable request.

## APPENDIX 1

### SEARCH STRATEGY

*PsycINFO (EBSCO) Search Terms*

AB OR TI: autis* OR asperger* OR ‘ASD’ OR neurodevelopmental OR DE ‘Autism Spectrum Disorders’ OR DE ‘Autistic Traits’

AND

AB OR TI: psychos* OR psychot* OR schizo* OR hallucinat* OR delusion*

OR DE ‘Psychosis’ OR DE ‘Affective Psychosis’ OR DE ‘Alcohol Induced Psychotic Disorders’ OR DE ‘Brief Psychotic Disorder’ OR DE ‘Capgras Syndrome’ OR DE ‘Childhood Onset Psychosis’ OR DE ‘Chronic Psychosis’ OR DE ‘Experimental Psychosis’ OR DE ‘Hallucinosis’ OR DE ‘Paranoid Psychosis’ OR DE ‘Postpartum Psychosis’ OR DE ‘Reactive Psychosis’ OR DE ‘Schizophrenia’ OR DE ‘Senile Dementia’ OR DE ‘Substance‐Induced Psychotic Disorders’ OR DE ‘Childhood Onset Psychosis’ OR DE ‘Childhood Onset Schizophrenia’ OR DE ‘Reactive Psychosis’ OR DE ‘Chronic Psychosis’ OR DE ‘Postpartum Psychosis’ OR DE ‘Paranoid Psychosis’ OR DE ‘Shared Paranoid Disorder’ OR DE ‘Brief Psychotic Disorder’ OR DE ‘Experimental Psychosis’ OR DE ‘Affective Psychosis’ OR DE ‘Process Schizophrenia’ OR DE ‘Substance‐Induced Psychotic Disorders’ OR DE ‘Alcohol Induced Psychotic Disorders’

OR DE ‘Schizophrenia’ OR DE ‘Acute Schizophrenia’ OR DE ‘Catatonic Schizophrenia’ OR DE ‘Childhood Onset Schizophrenia’ OR DE ‘Paranoid Schizophrenia’ OR DE ‘Process Schizophrenia’ OR DE ‘Schizoaffective Disorder’ OR DE ‘Schizophrenia (Disorganized Type)’ OR DE ‘Schizophreniform Disorder’ OR DE ‘Undifferentiated Schizophrenia’ OR DE ‘Schizophrenia (Disorganized Type)’ OR DE ‘Childhood Onset Schizophrenia’ OR DE ‘Undifferentiated Schizophrenia’ OR DE ‘Process Schizophrenia’ OR DE ‘Paranoid Schizophrenia’ OR DE ‘Fragmentation (Schizophrenia)’ OR DE ‘Catatonic Schizophrenia’ OR DE ‘Acute Schizophrenia’ OR DE ‘Schizophreniform Disorder’ OR DE ‘Schizotypy’ OR DE ‘Schizoaffective Disorder’

AND

AB OR TI: comorbid* OR co‐occur* OR concurrent OR prevalen* OR DE ‘Comorbidity’

*MEDLINE (EBSCO) Search Terms*

AB OR TI: autis* OR asperger* OR ‘ASD’ OR neurodevelopmental OR (MH ‘Autistic Disorder’) OR (MH ‘Autism Spectrum Disorder+’)

AND

AB OR TI: psychos* OR psychot* OR schizo* OR hallucinat* OR delusion* OR (MH ‘Psychotic Disorders+’) OR (MH ‘Schizophrenia Spectrum and Other Psychotic Disorders+’)

AND

AB OR TI: comorbid* OR co‐occur* OR concurrent OR prevalen* OR (MH ‘Comorbidity’)

*CINAHL (EBSCO) Search Terms*

AB OR TI: autis* OR asperger* OR ‘ASD’ OR neurodevelopmental OR (MH ‘Autistic Disorder’) OR (MH ‘Neurodiversity’) OR (MH ‘Developmental Disabilities’) OR (MH ‘Asperger Syndrome’)

AND

AB OR TI: psychos* OR psychot* OR schizo* OR hallucinat* OR delusion*

OR (MH ‘Postpartum Psychosis’) OR (MH ‘Psychoses, Substance‐Induced+’) OR (MH ‘ICU Psychosis’) OR (MH ‘Paranoid Disorders’) OR (MH ‘Psychoses, Alcoholic+’) OR (MH ‘Psychotic Disorders+’) OR (MH ‘Schizoaffective Disorder’) OR (MH ‘Affective Disorders, Psychotic+’) OR (MH ‘Organic Mental Disorders, Psychotic+’)

OR (MH ‘Schizotypal Personality Disorder’) OR (MH ‘Schizophrenia, Treatment‐Resistant’) OR (MH ‘Schizophrenia, Childhood’) OR (MH ‘Schizoaffective Disorder’) OR (MH ‘Schizophrenia+’)

AND

AB OR TI: comorbid* OR co‐occur* OR concurrent OR prevalen* OR (MH ‘Comorbidity’) OR (MH ‘Diagnosis, Dual (Psychiatry)’)

*Embase (Ovid) Search Terms*

AB OR TI: autis$ OR asperger$ OR ASD OR neurodevelopmental

AND

AB OR TI: psychos$ OR psychot$ OR schizo$ OR hallucinat$ OR delusion$

AND

AB OR TI: comorbid$ OR co‐occur$ OR concurrent OR prevalen$

- Limiter: Remove MEDLINE Records

*Web of Science Terms*

TI=(‘ autis*’ OR ‘asperger*’ OR ASD OR neurodevelopmental) OR AB=(‘autis*’ OR ‘asperger*’ OR ASD OR neurodevelopmental)

AND

TI=(‘psychos*’ OR ‘psychot*’ OR ‘schizo*’ OR ‘hallucinat*’ or ‘delusion*’) OR AB=(‘psychos*’ OR ‘psychot*’ OR ‘schizo*’ OR ‘hallucinat*’ or ‘delusion*’)

AND

TI=(‘comorbid*’ OR ‘co‐occur*’ OR ‘concurrent’ OR ‘prevalen*’) OR AB=(‘comorbid*’ OR ‘co‐occur*’ OR ‘concurrent’ OR ‘prevalen*’)

## References

[bjc70020-bib-0001] Abu‐Akel, A. , Apperly, I. , Spaniol, M. M. , Geng, J. J. , & Mevorach, C. (2018). Diametric effects of autism tendencies and psychosis proneness on attention control irrespective of task demands. Scientific Reports, 8(1), 8478. 10.1038/s41598-018-26821-7 29855492 PMC5981437

[bjc70020-bib-0002] Abu‐Akel, A. , Apperly, I. A. , Wood, S. J. , Hansen, P. C. , & Mevorach, C. (2017). Autism tendencies and psychosis proneness interactively modulate saliency cost. Schizophrenia Bulletin, 43(1), 142–151. 10.1093/schbul/sbw066 27217269 PMC5216849

[bjc70020-bib-0003] Abu‐Akel, A. M. , Apperly, I. A. , Wood, S. J. , & Hansen, P. C. (2017). Autism and psychosis expressions diametrically modulate the right temporoparietal junction. Social Neuroscience, 12(5), 506–518. 10.1080/17470919.2016.1190786 27187170

[bjc70020-bib-0004] American Psychiatric Association . (2013). Diagnostic and statistical manual of mental disorders (5th ed.). American Psychiatric Association. 10.1176/appi.books.9780890425596

[bjc70020-bib-0005] Amir, C. M. , Kapler, S. , Hoftman, G. D. , Kushan, L. , Zinberg, J. , Cadenhead, K. S. , Kennedy, L. , Cornblatt, B. A. , Keshavan, M. , Mathalon, D. H. , Perkins, D. O. , Stone, W. , Tsuang, M. T. , Walker, E. F. , Woods, S. W. , Cannon, T. D. , Addington, J. , & Bearden, C. E. (2023). Neurobehavioral risk factors influence prevalence and severity of hazardous substance use in youth at genetic and clinical high risk for psychosis. Frontiers in Psychiatry, 14, 1143315. 10.3389/fpsyt.2023.1143315 37151981 PMC10157227

[bjc70020-bib-0006] Baron‐Cohen, S. , Wheelwright, S. , Skinner, R. , Martin, J. , & Clubley, E. (2001). The autism‐Spectrum quotient (AQ): Evidence from Asperger syndrome/high‐functioning autism, males and females, scientists and mathematicians. Journal of Autism and Developmental Disorders, 31(1), 5–17. 10.1023/A:1005653411471 11439754

[bjc70020-bib-0007] Bin Dawood, A. , Dickinson, A. , & Jones, M. (2023). Investigation of the relationship between orientation discrimination thresholds, autistic, and schizotypal personality traits. International Journal of Cognitive Research in Science Engineering and Education, 11(3), 375–387. 10.23947/2334-8496-2023-11-3-375-387

[bjc70020-bib-0008] Blain, S. D. , Peterman, J. S. , & Park, S. (2017). Subtle cues missed: Impaired perception of emotion from gait in relation to schizotypy and autism spectrum traits. Schizophrenia Research, 183, 157–160. 10.1016/j.schres.2016.11.003 27838096

[bjc70020-bib-0009] Borenstein, M. , Hedges, L. V. , Higgins, J. P. T. , & Rothstein, H. R. (2009). Introduction to meta‐analysis (1st ed.). Wiley. 10.1002/9780470743386

[bjc70020-bib-0010] Butler, E. E. , Ward, R. , & Ramsey, R. (2015). Investigating the relationship between stable personality characteristics and automatic imitation. PLoS One, 10(6), e0129651. 10.1371/journal.pone.0129651 26079137 PMC4469457

[bjc70020-bib-0011] Cassidy, S. A. , Bradley, L. , Cogger‐Ward, H. , Shaw, R. , Bowen, E. , Glod, M. , Baron‐Cohen, S. , & Rodgers, J. (2020). Measurement properties of the suicidal behaviour questionnaire‐revised in autistic adults. Journal of Autism and Developmental Disorders, 50(10), 3477–3488. 10.1007/s10803-020-04431-5 32125569 PMC7502048

[bjc70020-bib-0012] CDC . (2023). Data and statistics on autism spectrum disorder. Centers for Disease Control and Prevention. https://www.cdc.gov/ncbddd/autism/data.html

[bjc70020-bib-0013] Chen, M. , Wei, H. , Chen, L. , Su, T. , Bai, Y. , Hsu, J. , Huang, K. , Chang, W. , Chen, T. , & Chen, Y. (2015). Autistic spectrum disorder, attention deficit hyperactivity disorder, and psychiatric comorbidities: A nationwide study. Research in Autism Spectrum Disorders, 10, 1–6. 10.1016/j.rasd.2014.10.014

[bjc70020-bib-0014] Choteau, L. , Raynal, P. , Goutaudier, N. , & Chabrol, H. (2016). Psychopathological traits in college students from top‐ranking french schools: Do autistic features impair success in science when associated with schizotypal traits? Psychiatry Research, 237, 218–223. 10.1016/j.psychres.2016.01.038 26809364

[bjc70020-bib-0015] Claridge, G. , & McDonald, A. (2009). An investigation into the relationships between convergent and divergent thinking, schizotypy, and autistic traits. Personality and Individual Differences, 46(8), 794–799. 10.1016/j.paid.2009.01.018

[bjc70020-bib-0016] Clark, J. , Glasziou, P. , Del Mar, C. , Bannach‐Brown, A. , Stehlik, P. , & Scott, A. M. (2020). A full systematic review was completed in 2 weeks using automation tools: A case study. Journal of Clinical Epidemiology, 121, 81–90. 10.1016/j.jclinepi.2020.01.008 32004673

[bjc70020-bib-0017] Conn, V. S. , Valentine, J. C. , Cooper, H. M. , & Rantz, M. J. (2003). Grey literature in meta‐analyses. Nursing Research, 52(4), 256–261. 10.1097/00006199-200307000-00008 12867783

[bjc70020-bib-0018] Constantino, J. N. , & Todd, R. D. (2003). Autistic traits in the general population: A twin study. Archives of General Psychiatry, 60(5), 524–530. 10.1001/archpsyc.60.5.524 12742874

[bjc70020-bib-0019] Craig, J. S. , Hatton, C. , Craig, F. B. , & Bentall, R. P. (2004). Persecutory beliefs, attributions and theory of mind: Comparison of patients with paranoid delusions, Asperger's syndrome and healthy controls. Schizophrenia Research, 69(1), 29–33. 10.1016/S0920-9964(03)00154-3 15145468

[bjc70020-bib-0020] Dardani, C. , Schalbroeck, R. , Madley‐Dowd, P. , Jones, H. J. , Strelchuk, D. , Hammerton, G. , Croft, J. , Sullivan, S. A. , Zammit, S. , Selten, J.‐P. , & Rai, D. (2023). Childhood trauma As a mediator of the association between autistic traits and psychotic experiences: Evidence from the Avon longitudinal study of parents and children cohort. Schizophrenia Bulletin, 49(2), 364–374. 10.1093/schbul/sbac167 36434745 PMC10016398

[bjc70020-bib-0021] Davies, C. , Segre, G. , Estradé, A. , Radua, J. , De Micheli, A. , Provenzani, U. , Oliver, D. , Salazar De Pablo, G. , Ramella‐Cravaro, V. , Besozzi, M. , Dazzan, P. , Miele, M. , Caputo, G. , Spallarossa, C. , Crossland, G. , Ilyas, A. , Spada, G. , Politi, P. , Murray, R. M. , … Fusar‐Poli, P. (2020). Prenatal and perinatal risk and protective factors for psychosis: A systematic review and meta‐analysis. The Lancet Psychiatry, 7(5), 399–410. 10.1016/S2215-0366(20)30057-2 32220288

[bjc70020-bib-0022] Davignon, M. N. , Qian, Y. , Massolo, M. , & Croen, L. A. (2018). Psychiatric and medical conditions in transition‐aged individuals with ASD. Pediatrics, 141(Suppl 4), S335–S345. 10.1542/peds.2016-4300K 29610415

[bjc70020-bib-0023] de Bildt, A. , Sytema, S. , Meffert, H. , & Bastiaansen, J. A. C. J. (2016). The autism diagnostic observation schedule, module 4: Application of the revised algorithms in an independent, well‐defined, Dutch sample (n=93). Journal of Autism and Developmental Disorders, 46(1), 21–30. 10.1007/s10803-015-2532-4 26319249 PMC4706589

[bjc70020-bib-0024] De Crescenzo, F. , Postorino, V. , Siracusano, M. , Riccioni, A. , Armando, M. , Curatolo, P. , & Mazzone, L. (2019). Autistic symptoms in schizophrenia spectrum disorders: A systematic review and meta‐analysis. Frontiers in Psychiatry, 10, 78. 10.3389/fpsyt.2019.00078 30846948 PMC6393379

[bjc70020-bib-0025] Debbane, M. , Eliez, S. , Badoud, D. , Conus, P. , Fluckiger, R. , & Schultze‐Lutter, F. (2015). Developing psychosis and its risk states through the lens of Schizotypy. Schizophrenia Bulletin, 41(suppl 2), S396–S407. 10.1093/schbul/sbu176 25548386 PMC4373628

[bjc70020-bib-0026] Del Giudice, M. , Angeleri, R. , Brizio, A. , & Elena, M. R. (2010). The evolution of autistic‐like and schizotypal traits: A sexual selection hypothesis. Frontiers in Psychology, 1, 41. 10.3389/fpsyg.2010.00041 21833210 PMC3153759

[bjc70020-bib-0027] Del Giudice, M. , Klimczuk, A. C. E. , Traficonte, D. M. , & Maestripieri, D. (2014). Autistic‐like and schizotypal traits in a life history perspective: Diametrical associations with impulsivity, sensation seeking, and sociosexual behavior. Evolution and Human Behavior, 35(5), 415–424. 10.1016/j.evolhumbehav.2014.05.007

[bjc70020-bib-0028] Dinsdale, N. L. , Hurd, P. L. , Wakabayashi, A. , Elliot, M. , & Crespi, B. J. (2013). How are autism and Schizotypy related? Evidence from a non‐clinical population. PLoS One, 8(5), e63316. 10.1371/journal.pone.0063316 23691021 PMC3655150

[bjc70020-bib-0029] Dossetor, D. R. (2007). “All that glitters is not gold”: Misdiagnosis of psychosis in pervasive developmental disorders—A case series. Clinical Child Psychology and Psychiatry, 12(4), 537–548. 10.1177/1359104507078476 18095536

[bjc70020-bib-0030] Downes, M. J. , Brennan, M. L. , Williams, H. C. , & Dean, R. S. (2016). Development of a critical appraisal tool to assess the quality of cross‐sectional studies (AXIS). BMJ Open, 6(12), e011458. 10.1136/bmjopen-2016-011458 PMC5168618 27932337

[bjc70020-bib-0031] Fendrich, S. J. , Koralnik, L. R. , Bonner, M. , Goetz, D. , Joe, P. , Lee, J. , Mueller, B. , Robinson‐Papp, J. , Gonen, O. , Clemente, J. C. , & Malaspina, D. (2022). Patient‐reported exposures and outcomes link the gut‐brain axis and inflammatory pathways to specific symptoms of severe mental illness. Psychiatry Research, 312, 114526. 10.1016/j.psychres.2022.114526 35462090

[bjc70020-bib-0032] Fleischman, R. , Wilson, C. , Demro, C. , Jameson, N. , Anthony, L. , & Schiffman, J. (2016). *Analysis of psychosis risk interview questions for individuals with autistic spectrum disorders*. The 20th Annual Undergraduate Research and Creative Achievement Day, Baltimore, MD.

[bjc70020-bib-0033] Ford, T. C. , Apputhurai, P. , Meyer, D. , & Crewther, D. P. (2017). Confirmatory factor analysis of autism and schizophrenia spectrum traits. Personality and Individual Differences, 110, 80–84. 10.1016/j.paid.2017.01.033

[bjc70020-bib-0034] Gardener, H. , Spiegelman, D. , & Buka, S. L. (2009). Prenatal risk factors for autism: Comprehensive meta‐analysis. British Journal of Psychiatry, 195(1), 7–14. 10.1192/bjp.bp.108.051672 PMC3712619 19567888

[bjc70020-bib-0035] Gardener, H. , Spiegelman, D. , & Buka, S. L. (2011). Perinatal and neonatal risk factors for autism: A comprehensive meta‐analysis. Pediatrics, 128(2), 344–355. 10.1542/peds.2010-1036 21746727 PMC3387855

[bjc70020-bib-0036] Georgiou, N. , Delfabbro, P. , & Balzan, R. (2021). Autistic traits as a potential confounding factor in the relationship between schizotypy and conspiracy beliefs. Cognitive Neuropsychiatry, 26(4), 273–292. 10.1080/13546805.2021.1924650 33970807

[bjc70020-bib-0037] Gesi, C. , Giacovelli, L. , Reibman, Y. L. , & Dell'Osso, B. (2024). Beyond imagination: Sorting out and treating psychosis in the context of autism spectrum disorder. Journal of Psychiatric Research, 173, 363–366. 10.1016/j.jpsychires.2024.03.043 38593694

[bjc70020-bib-0038] Gillespie, S. M. , Mitchell, I. J. , & Abu‐Akel, A. M. (2017). Autistic traits and positive psychotic experiences modulate the association of psychopathic tendencies with theory of mind in opposite directions. Scientific Reports, 7(1), 6485. 10.1038/s41598-017-06995-2 28743994 PMC5526986

[bjc70020-bib-0039] Gong, J.‐B. , Wang, Y. , Lui, S. S. Y. , Cheung, E. F. C. , & Chan, R. C. K. (2017). Childhood trauma is not a confounder of the overlap between autistic and schizotypal traits: A study in a non‐clinical adult sample. Psychiatry Research, 257, 111–117. 10.1016/j.psychres.2017.07.035 28750214

[bjc70020-bib-0040] Grigoriadis, S. , & Seeman, M. V. (2002). The role of estrogen in schizophrenia: Implications for schizophrenia practice guidelines for women. The Canadian Journal of Psychiatry, 47(5), 437–442. 10.1177/070674370204700504 12085678

[bjc70020-bib-0041] Hand, B. N. , Angell, A. M. , Harris, L. , & Carpenter, L. A. (2020). Prevalence of physical and mental health conditions in Medicare‐enrolled, autistic older adults. Autism: The International Journal of Research & Practice, 24(3), 755–764. 10.1177/1362361319890793 31773968 PMC7433648

[bjc70020-bib-0042] Helverschou, S. , Bakken, T. , & Martinsen, H. (2009). The psychopathology in autism checklist (PAC): A pilot study. Research in Autism Spectrum Disorders, 3(1), 179–195. 10.1016/j.rasd.2008.05.004

[bjc70020-bib-0043] Horder, J. , Wilson, C. E. , Mendez, M. A. , & Murphy, D. G. (2014). Autistic traits and abnormal sensory experiences in adults. Journal of Autism and Developmental Disorders, 44(6), 1461–1469. 10.1007/s10803-013-2012-7 24305777 PMC4022987

[bjc70020-bib-0044] Houghton, R. , Liu, C. , & Bolognani, F. (2018). Psychiatric comorbidities and psychotropic medication use in autism: A matched cohort study with ADHD and general population comparator groups in the United Kingdom. Autism Research, 11(12), 1690–1700. 10.1002/aur.2040 30380202

[bjc70020-bib-0045] Hurst, R. M. , Nelson‐Gray, R. O. , Mitchell, J. T. , & Kwapil, T. R. (2007). The relationship of Asperger's characteristics and schizotypal personality traits in a non‐clinical adult sample. Journal of Autism and Developmental Disorders, 37(9), 1711–1720. 10.1007/s10803-006-0302-z 17149668

[bjc70020-bib-0046] Issac, A. , Halemani, K. , Shetty, A. , Thimmappa, L. , Vijay, V. , Koni, K. , Mishra, P. , & Kapoor, V. (2025). The global prevalence of autism spectrum disorder in children: A systematic review and meta‐analysis. Osong Public Health and Research Perspectives, 16(1), 3–27. 10.24171/j.phrp.2024.0286 39933560 PMC11917377

[bjc70020-bib-0047] Kamp‐Becker, I. (2024). Autism spectrum disorder in ICD‐11—A critical reflection of its possible impact on clinical practice and research. Molecular Psychiatry, 29(3), 633–638. 10.1038/s41380-023-02354-y 38273107 PMC11153155

[bjc70020-bib-0048] Karvelis, P. , Seitz, A. R. , Lawrie, S. M. , & Seriès, P. (2018). Autistic traits, but not schizotypy, predict increased weighting of sensory information in Bayesian visual integration. eLife, 7, e34115. 10.7554/eLife.34115 29757142 PMC5966274

[bjc70020-bib-0049] Kendler, K. S. , Ohlsson, H. , Sundquist, J. , & Sundquist, K. (2024). The genetic epidemiology of schizotypal personality disorder. Psychological Medicine, 54(9), 2144–2151. 10.1017/S0033291724000230 38362845 PMC11413339

[bjc70020-bib-0050] Kincaid, D. , Doris, M. , Shannon, C. , & Mulholland, C. (2017). What is the prevalence of autism spectrum disorder and ASD traits in psychosis? A systematic review. Psychiatry Research, 250, 99–105. 10.1016/j.psychres.2017.01.017 28152400

[bjc70020-bib-0051] Kirkbride, J. B. , Errazuriz, A. , Croudace, T. J. , Morgan, C. , Jackson, D. , Boydell, J. , Murray, R. M. , & Jones, P. B. (2012). Incidence of schizophrenia and other psychoses in England, 1950–2009: A systematic review and meta‐analyses. PLoS One, 7(3), e31660. 10.1371/journal.pone.0031660 22457710 PMC3310436

[bjc70020-bib-0052] Kiyono, T. , Morita, M. , Morishima, R. , Fujikawa, S. , Yamasaki, S. , Nishida, A. , Ando, S. , & Kasai, K. (2020). The prevalence of psychotic experiences in autism Spectrum disorder and autistic traits: A systematic review and meta‐analysis. Schizophrenia Bulletin Open, 1(1), sgaa046. 10.1093/schizbullopen/sgaa046

[bjc70020-bib-0053] Kohane, I. , McMurry, A. , Weber, G. , MacFadden, D. , Rappaport, L. , Kunkel, L. , Bickel, J. , Wattanasin, N. , Spence, S. , Murphy, S. , & Churchill, S. (2012). The Co‐morbidity burden of children and young adults with autism Spectrum disorders. PLoS One, 7(4), e33224. 10.1371/journal.pone.0033224 22511918 PMC3325235

[bjc70020-bib-0054] Krieger, I. , Grossman‐Giron, A. , Comaneshter, D. , Weinstein, O. , Kridin, K. , Cohen, A. D. , & Tzur Bitan, D. (2021). The co‐occurrence of autistic spectrum disorder and schizophrenia: A nationwide population‐based study. Journal of Psychiatric Research, 138, 280–283. 10.1016/j.jpsychires.2021.04.012 33872965

[bjc70020-bib-0055] Lai, M. , Kassee, C. , Besney, R. , Bonato, S. , Hull, L. , Mandy, W. , Szatmari, P. , & Ameis, S. (2019). Prevalence of co‐occurring mental health diagnoses in the autism population: A systematic review and meta‐analysis. The Lancet Psychiatry, 6(10), 819–829. 10.1016/S2215-0366(19)30289-5 31447415

[bjc70020-bib-0056] Larson, F. V. , Wagner, A. P. , Jones, P. B. , Tantam, D. , Lai, M.‐C. , Baron‐Cohen, S. , & Holland, A. J. (2017). Psychosis in autism: Comparison of the features of both conditions in a dually affected cohort. The British Journal of Psychiatry, 210(4), 269–275. 10.1192/bjp.bp.116.187682 27979819 PMC5376719

[bjc70020-bib-0057] Li, R. , Ma, X. , Wang, G. , Yang, J. , & Wang, C. (2016). Why sex differences in schizophrenia? Journal of Translational Neuroscience, 1(1), 37–42. 29152382 PMC5688947

[bjc70020-bib-0058] Linscott, R. J. , & Van Os, J. (2013). An updated and conservative systematic review and meta‐analysis of epidemiological evidence on psychotic experiences in children and adults: On the pathway from proneness to persistence to dimensional expression across mental disorders. Psychological Medicine, 43(6), 1133–1149. 10.1017/S0033291712001626 22850401

[bjc70020-bib-0059] Louzolo, A. , Gustavsson, P. , Tigerstrom, L. , Ingvar, M. , Olsson, A. , & Petrovic, P. (2017). Delusion‐proneness displays comorbidity with traits of autistic‐spectrum disorders and ADHD. PLoS One, 12(5), e0177820. 10.1371/journal.pone.0177820 28542365 PMC5436821

[bjc70020-bib-0060] Lugnegård, T. , Hallerbäck, M. U. , & Gillberg, C. (2015). Asperger syndrome and schizophrenia: Overlap of self‐reported autistic traits using the autism‐spectrum quotient (AQ). Nordic Journal of Psychiatry, 69(4), 268–274. 10.3109/08039488.2014.972452 25389915

[bjc70020-bib-0061] Lugo Marín, J. , Alviani Rodríguez‐Franco, M. , Mahtani Chugani, V. , Magán Maganto, M. , Díez Villoria, E. , & Canal Bedia, R. (2018). Prevalence of schizophrenia Spectrum disorders in average‐IQ adults with autism Spectrum disorders: A meta‐analysis. Journal of Autism and Developmental Disorders, 48(1), 239–250. 10.1007/s10803-017-3328-5 28980099

[bjc70020-bib-0062] Mamah, D. , Mutiso, V. , Gitonga, I. , Tele, A. , & Ndetei, D. M. (2022). A population‐based survey of autistic traits in Kenyan adolescents and young adults. South African Journal of Psychiatry, 28((Mamah) Department of Psychiatry, Washington University, St. Louis, United States), a1694. 10.4102/sajpsychiatry.v28i0.1694 PMC8905436 35281966

[bjc70020-bib-0063] Martinez, A. P. , Wickham, S. , Rowse, G. , Milne, E. , & Bentall, R. P. (2021). Robust association between autistic traits and psychotic‐like experiences in the adult general population: Epidemiological study from the 2007 Adult Psychiatric Morbidity Survey and replication with the 2014 APMS. Psychological Medicine, 51(15), 2707–2713. 10.1017/S0033291720001373 32441234

[bjc70020-bib-0064] Martinez, G. , Alexandre, C. , Mam‐Lam‐Fook, C. , Bendjemaa, N. , Gaillard, R. , Garel, P. , Dziobek, I. , Amado, I. , & Krebs, M.‐O. (2017). Phenotypic continuum between autism and schizophrenia: Evidence from the movie for the assessment of social cognition (MASC). Schizophrenia Research, 185, 161–166. 10.1016/j.schres.2017.01.012 28089135

[bjc70020-bib-0065] Matsuo, J. , Kamio, Y. , Takahashi, H. , Ota, M. , Teraishi, T. , Hori, H. , Nagashima, A. , Takei, R. , Higuchi, T. , Motohashi, N. , & Kunugi, H. (2015). Autistic‐like traits in adult patients with mood disorders and schizophrenia. PLoS One, 10(4), e0122711. 10.1371/journal.pone.0122711 25838109 PMC4383414

[bjc70020-bib-0066] Mawn, L. , Campbell, T. , Aynsworth, C. , Beckwith, H. , Luce, A. , Barclay, N. , Dodgson, G. , & Freeston, M. H. (2020). Comorbidity of obsessive‐compulsive and psychotic experiences: A systematic review and meta‐analysis. Journal of Obsessive‐Compulsive and Related Disorders, 26, 100539. 10.1016/j.jocrd.2020.100539

[bjc70020-bib-0067] McAuley, L. , Pham, B. , Tugwell, P. , & Moher, D. (2000). Does the inclusion of grey literature influence estimates of intervention effectiveness reported in meta‐analyses? Lancet (London, England), 356(9237), 1228–1231. 10.1016/S0140-6736(00)02786-0 11072941

[bjc70020-bib-0068] McGrath, J. J. , Saha, S. , Al‐Hamzawi, A. , Alonso, J. , Bromet, E. J. , Bruffaerts, R. , Caldas‐de‐Almeida, J. M. , Chiu, W. T. , De Jonge, P. , Fayyad, J. , Florescu, S. , Gureje, O. , Haro, J. M. , Hu, C. , Kovess‐Masfety, V. , Lepine, J. P. , Lim, C. C. W. , Mora, M. E. M. , Navarro‐Mateu, F. , … Kessler, R. C. (2015). Psychotic experiences in the general population: A cross‐national analysis based on 31 261 respondents from 18 countries. JAMA Psychiatry, 72(7), 697. 10.1001/jamapsychiatry.2015.0575 26018466 PMC5120396

[bjc70020-bib-0069] Mealey, A. , Abbott, G. , Byrne, L. K. , & McGillivray, J. (2014). Overlap between autistic and schizotypal personality traits is not accounted for by anxiety and depression. Psychiatry Research, 219(2), 380–385. 10.1016/j.psychres.2014.05.040 24930576

[bjc70020-bib-0070] Melchers, M. , Montag, C. , Markett, S. , & Reuter, M. (2015). Assessment of empathy via self‐report and behavioural paradigms: Data on convergent and discriminant validity. Cognitive Neuropsychiatry, 20(2), 157–171. 10.1080/13546805.2014.991781 25530230

[bjc70020-bib-0071] Milne, E. , Dickinson, A. , & Smith, R. (2017). Adults with autism spectrum conditions experience increased levels of anomalous perception. PLoS One, 12(5), e0177804. 10.1371/journal.pone.0177804 28542171 PMC5436824

[bjc70020-bib-0072] Moreno‐Küstner, B. , Martín, C. , & Pastor, L. (2018). Prevalence of psychotic disorders and its association with methodological issues. A systematic review and meta‐analyses. PLoS One, 13(4), e0195687. 10.1371/journal.pone.0195687 29649252 PMC5896987

[bjc70020-bib-0073] Nair, A. , & Borkar, N. (2023). Significance of including grey literature search in systematic reviews and meta‐analyses. Saudi Journal of Anesthesia, 17(2), 295–296. 10.4103/sja.sja_635_22 37260633 PMC10228866

[bjc70020-bib-0074] Nakamura, D. , Hanawa, Y. , Seki, S. , Yamauchi, M. , Iwami, Y. , Nagatsuka, Y. , Suzuki, H. , Aoyagi, K. , Hayashi, W. , Otowa, T. , & Iwanami, A. (2024). Predictive model using autism diagnostic observation schedule, second edition for differential diagnosis between schizophrenia and autism spectrum disorder. Frontiers in Psychiatry, 15, 1493158. 10.3389/fpsyt.2024.1493158 39744545 PMC11689654

[bjc70020-bib-0075] Nenadić, I. , Meller, T. , Evermann, U. , Schmitt, S. , Pfarr, J.‐K. , Abu‐Akel, A. , & Grezellschak, S. (2021). Subclinical schizotypal vs. autistic traits show overlapping and diametrically opposed facets in a non‐clinical population. Schizophrenia Research, 231, 32–41. 10.1016/j.schres.2021.02.018 33744683

[bjc70020-bib-0076] Ouzzani, M. , Hammady, H. , Fedorowicz, Z. , & Elmagarmid, A. (2016). Rayyan—A web and mobile app for systematic reviews. Systematic Reviews, 5(1), 210. 10.1186/s13643-016-0384-4 27919275 PMC5139140

[bjc70020-bib-0077] Page, M. J. , Moher, D. , Bossuyt, P. M. , Boutron, I. , Hoffmann, T. C. , Mulrow, C. D. , Shamseer, L. , Tetzlaff, J. M. , Akl, E. A. , Brennan, S. E. , Chou, R. , Glanville, J. , Grimshaw, J. M. , Hróbjartsson, A. , Lalu, M. M. , Li, T. , Loder, E. W. , Mayo‐Wilson, E. , McDonald, S. , … McKenzie, J. E. (2021). PRISMA 2020 explanation and elaboration: Updated guidance and exemplars for reporting systematic reviews. BMJ, 372, n160. 10.1136/bmj.n160 33781993 PMC8005925

[bjc70020-bib-0078] Parvaiz, R. , Vindbjerg, E. , Crespi, B. , Happe, F. , Schalbroeck, R. , Al‐Sayegh, Z. , Danielsen, I.‐M. , Tonge, B. , Videbech, P. , & Abu‐Akel, A. (2023). Protocol for the development and testing of the schiZotypy autism questionnaire (ZAQ) in adults: A new screening tool to discriminate autism spectrum disorder from schizotypal disorder. BMC Psychiatry, 23(1), 200. 10.1186/s12888-023-04690-3 36978026 PMC10044373

[bjc70020-bib-0079] Perälä, J. , Suvisaari, J. , Saarni, S. I. , Kuoppasalmi, K. , Isometsä, E. , Pirkola, S. , Partonen, T. , Tuulio‐Henriksson, A. , Hintikka, J. , Kieseppä, T. , Härkänen, T. , Koskinen, S. , & Lönnqvist, J. (2007). Lifetime prevalence of psychotic and bipolar I disorders in a general population. Archives of General Psychiatry, 64(1), 19. 10.1001/archpsyc.64.1.19 17199051

[bjc70020-bib-0080] Pinkham, A. E. , Hopfinger, J. B. , Pelphrey, K. A. , Piven, J. , & Penn, D. L. (2008). Neural bases for impaired social cognition in schizophrenia and autism spectrum disorders. Schizophrenia Research, 99(1–3), 164–175. 10.1016/j.schres.2007.10.024 18053686 PMC2740744

[bjc70020-bib-0081] Raine, A. (1991). The SPQ: A scale for the assessment of schizotypal personality based on DSM‐III‐R criteria. Schizophrenia Bulletin, 17(4), 555–564. 10.1093/schbul/17.4.555 1805349

[bjc70020-bib-0082] Rapoport, J. , Chavez, A. , Greenstein, D. , Addington, A. , & Gogtay, N. (2009). Autism Spectrum disorders and childhood‐onset schizophrenia: Clinical and biological contributions to a relation revisited. Journal of the American Academy of Child and Adolescent Psychiatry, 48(1), 10–18. 10.1097/CHI.0b013e31818b1c63 19218893 PMC2664646

[bjc70020-bib-0083] Raynal, P. , Goutaudier, N. , Nidetch, V. , & Chabrol, H. (2016). Typology of schizotypy in non‐clinical young adults: Psychopathological and personality disorder traits correlates. Psychiatry Research, 246, 182–187. 10.1016/j.psychres.2016.09.042 27718467

[bjc70020-bib-0084] Raynal, P. , Melioli, T. , Goutaudier, N. , & Chabrol, H. (2016). Is the link between autistic traits and ability to succeed in science independent of other psychopathological dimensions? European Review Of Applied Psychology‐Revue Europeenne De Psychologie Appliquee, 66(6), 301–307. 10.1016/j.erap.2016.05.004

[bjc70020-bib-0085] Rees, E. , Creeth, H. D. J. , Hwu, H.‐G. , Chen, W. J. , Tsuang, M. , Glatt, S. J. , Rey, R. , Kirov, G. , Walters, J. T. R. , Holmans, P. , Owen, M. J. , & O'Donovan, M. C. (2021). Schizophrenia, autism spectrum disorders and developmental disorders share specific disruptive coding mutations. Nature Communications, 12(1), 5353. 10.1038/s41467-021-25532-4 PMC8429694 34504065

[bjc70020-bib-0086] Roy, M. , Prox‐Vagedes, V. , Ohlmeier, M. D. , & Dillo, W. (2015). Beyond childhood: Psychiatric comorbidities and social background of adults with Asperger syndrome. Psychiatria Danubina, 27(1), 50–59. psyh. 25751431

[bjc70020-bib-0087] Russell‐Smith, S. N. , Bayliss, D. M. , & Maybery, M. T. (2013). Unique sets of social and mood characteristics differentiate autistic and negative schizotypy traits in a young adult non‐clinical sample. Personality and Individual Differences, 55(5), 542–546. 10.1016/j.paid.2013.04.030

[bjc70020-bib-0088] Russell‐Smith, S. N. , Maybery, M. T. , & Bayliss, D. M. (2011). Relationships between autistic‐like and schizotypy traits: An analysis using the autism Spectrum quotient and Oxford‐Liverpool inventory of feelings and experiences. Personality and Individual Differences, 51(2), 128–132. 10.1016/j.paid.2011.03.027

[bjc70020-bib-0089] Ruzich, E. , Allison, C. , Smith, P. , Watson, P. , Auyeung, B. , Ring, H. , & Baron‐Cohen, S. (2015). Measuring autistic traits in the general population: A systematic review of the autism‐Spectrum quotient (AQ) in a nonclinical population sample of 6,900 typical adult males and females. Molecular Autism, 6(1), 2. 10.1186/2040-2392-6-2 25874074 PMC4396128

[bjc70020-bib-0090] Salazar De Pablo, G. , Radua, J. , Pereira, J. , Bonoldi, I. , Arienti, V. , Besana, F. , Soardo, L. , Cabras, A. , Fortea, L. , Catalan, A. , Vaquerizo‐Serrano, J. , Coronelli, F. , Kaur, S. , Da Silva, J. , Shin, J. I. , Solmi, M. , Brondino, N. , Politi, P. , McGuire, P. , & Fusar‐Poli, P. (2021). Probability of transition to psychosis in individuals at clinical high risk: An updated meta‐analysis. JAMA Psychiatry, 78(9), 970–978. 10.1001/jamapsychiatry.2021.0830 34259821 PMC8281006

[bjc70020-bib-0091] Salazar De Pablo, G. , Woods, S. W. , Drymonitou, G. , De Diego, H. , & Fusar‐Poli, P. (2021). Prevalence of individuals at clinical high‐risk of psychosis in the general population and clinical samples: Systematic review and meta‐analysis. Brain Sciences, 11(11), 1544. 10.3390/brainsci11111544 34827543 PMC8615691

[bjc70020-bib-0092] Salminen, I. , Read, S. , & Crespi, B. (2022). Do the diverse phenotypes of Prader‐Willi syndrome reflect extremes of covariation in typical populations. Frontiers in Genetics, 13, 1041943. 10.3389/fgene.2022.1041943 36506301 PMC9731222

[bjc70020-bib-0093] Samadi, S. A. , Mahmoodizadeh, A. , & McConkey, R. (2012). A national study of the prevalence of autism among five‐year‐old children in Iran. Autism, 16(1), 5–14. 10.1177/1362361311407091 21610190

[bjc70020-bib-0094] Sampson, K. N. , Upthegrove, R. , Abu‐Akel, A. , Haque, S. , Wood, S. J. , & Reniers, R. (2021). Co‐occurrence of autistic and psychotic traits: Implications for depression, self‐harm and suicidality. Psychological Medicine, 51(8), 1364–1372. 10.1017/S0033291720000124 32081111

[bjc70020-bib-0095] Sasamoto, A. , Miyata, J. , Hirao, K. , Fujiwara, H. , Kawada, R. , Fujimoto, S. , Tanaka, Y. , Kubota, M. , Sawamoto, N. , Fukuyama, H. , Takahashi, H. , & Murai, T. (2011). Social impairment in schizophrenia revealed by autism‐Spectrum quotient correlated with gray matter reduction. Social Neuroscience, 6(5–6), 548–558. 10.1080/17470919.2011.575693 21943127

[bjc70020-bib-0096] Schendel, D. E. , Overgaard, M. , Christensen, J. , Hjort, L. , Jorgensen, M. , Vestergaard, M. , & Parner, E. T. (2016). Association of psychiatric and neurologic comorbidity with mortality among persons with autism spectrum disorder in a Danish population. JAMA Pediatrics, 170(3), 243–250. 10.1001/jamapediatrics.2015.3935 26752506

[bjc70020-bib-0097] Sebat, J. , Levy, D. L. , & McCarthy, S. E. (2009). Rare structural variants in schizophrenia: One disorder, multiple mutations; one mutation, multiple disorders. Trends in Genetics, 25(12), 528–535. 10.1016/j.tig.2009.10.004 19883952 PMC3351381

[bjc70020-bib-0098] Shi, L. , Liu, W. , Shi, H. , Yan, C. , Wang, Y. , Wang, Y. , Cheung, E. F. C. , & Chan, R. C. K. (2017). Co‐occurrence of autistic and schizotypal traits and its association with emotional and psychosocial function in Chinese college students. Psychiatry Research, 248, 64–70. 10.1016/j.psychres.2016.12.021 28024179

[bjc70020-bib-0099] Sierro, G. , Rossier, J. , & Mohr, C. (2016). Validation of the French Autism Spectrum Quotient scale and its relationships with schizotypy and Eysenckian personality traits. Comprehensive Psychiatry, 68, 147–155. 10.1016/j.comppsych.2016.03.011 27234196

[bjc70020-bib-0100] Solberg, B. S. , Zayats, T. , Posserud, M.‐B. , Halmøy, A. , Engeland, A. , Haavik, J. , & Klungsøyr, K. (2019b). Patterns of psychiatric comorbidity and genetic correlations provide new insights into differences between attention‐deficit/hyperactivity disorder and autism spectrum disorder. Biological Psychiatry, 86(8), 587–598. 10.1016/j.biopsych.2019.04.021 31182215 PMC6764861

[bjc70020-bib-0102] Starling, J. , & Dossetor, D. (2009). Pervasive developmental disorders and psychosis. Current Psychiatry Reports, 11(3), 190–196. 10.1007/s11920-009-0030-0 19470280

[bjc70020-bib-0103] Stralin, P. , & Hetta, J. (2021). First episode psychosis: Register‐based study of comorbid psychiatric disorders and medications before and after. European Archives of Psychiatry and Clinical Neuroscience, 271(2), 303–313. 10.1007/s00406-020-01139-6 32458108 PMC7960599

[bjc70020-bib-0104] Suen, Y. N. , Chau, A. P. Y. , Wong, S. M. Y. , Hui, C. L. M. , Chan, S. K. W. , Lee, E. H. M. , Wong, M. T. H. , & Chen, E. Y. H. (2024). Comorbidity of autism spectrum and attention deficit/hyperactivity disorder symptoms and their associations with 1‐year mental health outcomes in adolescents and young adults. Psychiatry Research, 331, 115657. 10.1016/j.psychres.2023.115657 38056129

[bjc70020-bib-0105] Sullivan, S. , Rai, D. , Golding, J. , Zammit, S. , & Steer, C. (2013). The association between autism spectrum disorder and psychotic experiences in the Avon longitudinal study of parents and children (ALSPAC) birth cohort. Journal of the American Academy of Child and Adolescent Psychiatry, 52(8), 806–814.e2. 10.1016/j.jaac.2013.05.010 23880491

[bjc70020-bib-0106] Supekar, K. , Iyer, T. , & Menon, V. (2017). The influence of sex and age on prevalence rates of comorbid conditions in autism. Autism Research, 10(5), 778–789. 10.1002/aur.1741 28188687

[bjc70020-bib-0107] Talantseva, O. I. , Romanova, R. S. , Shurdova, E. M. , Dolgorukova, T. A. , Sologub, P. S. , Titova, O. S. , Kleeva, D. F. , & Grigorenko, E. L. (2023). The global prevalence of autism spectrum disorder: A three‐level meta‐analysis. Frontiers in Psychiatry, 14, 1071181. 10.3389/fpsyt.2023.1071181 36846240 PMC9947250

[bjc70020-bib-0108] Tint, A. , Chung, H. , Lai, M.‐C. , Balogh, R. , Lin, E. , Durbin, A. , & Lunsky, Y. (2023). Health conditions and service use of autistic women and men: A retrospective population‐based case–control study. Autism, 27(6), 1641–1657. 10.1177/13623613221144353 36588296 PMC10374994

[bjc70020-bib-0109] Underwood, J. F. G. , DelPozo‐Banos, M. , Frizzati, A. , Rai, D. , John, A. , & Hall, J. (2022). Neurological and psychiatric disorders among autistic adults: A population healthcare record study. Psychological Medicine, 53, 5663–5673. 10.1017/S0033291722002884 36189783 PMC10482712

[bjc70020-bib-0110] Upthegrove, R. , Abu‐Akel, A. , Chisholm, K. , Lin, A. , Zahid, S. , Pelton, M. , Apperly, I. , Hansen, P. , & Wood, S. (2018). Autism and psychosis: Clinical implications for depression and suicide. Schizophrenia Research, 195, 80–85. 10.1016/j.schres.2017.08.028 28823724

[bjc70020-bib-0111] Vaquerizo‐Serrano, J. , Salazar De Pablo, G. , Singh, J. , & Santosh, P. (2022). Autism spectrum disorder and clinical high risk for psychosis: A systematic review and meta‐analysis. Journal of Autism and Developmental Disorders, 52(4), 1568–1586. 10.1007/s10803-021-05046-0 33993403 PMC8938385

[bjc70020-bib-0112] Varcin, K. , Herniman, S. , Lin, A. , Chen, Y. , Perry, Y. , Pugh, C. , Chisholm, K. , Whitehouse, A. , & Wood, S. (2022). Occurrence of psychosis and bipolar disorder in adults with autism: A systematic review and meta‐analysis. Neuroscience and Biobehavioral Reviews, 134, 104543. 10.1016/j.neubiorev.2022.104543 35063494

[bjc70020-bib-0113] Vohra, R. , Madhavan, S. , & Sambamoorthi, U. (2017). Comorbidity prevalence, healthcare utilization, and expenditures of Medicaid enrolled adults with autism spectrum disorders. Autism, 21(8), 995–1009. 10.1177/1362361316665222 27875247 PMC5517354

[bjc70020-bib-0114] Wakabayashi, A. , Baron‐Cohen, S. , & Ashwin, C. (2012). Do the traits of autism‐spectrum overlap with those of schizophrenia or obsessive‐compulsive disorder in the general population? Research in Autism Spectrum Disorders, 6(2), 717–725. 10.1016/j.rasd.2011.09.008

[bjc70020-bib-0115] Wilson, C. S. (2018). *Feasibility of psychosis risk assessment for individuals diagnosed with an autism spectrum disorder: A cognitive exploration of Item Interpretation of the Structured Interview for psychosis‐risk syndromes* (2018‐09132‐116; Issues 3‐B(E)) [ProQuest Information & Learning]. https://search.ebscohost.com/login.aspx?direct=true&db=psyh&AN=2018‐09132‐116&site=ehost‐live

[bjc70020-bib-0116] Wilson, C. S. , Anthony, L. , Kenworthy, L. , Fleischman, R. , Demro, C. , Andorko, N. , Chelsea Armour, A. , & Schiffman, J. (2020). Feasibility of psychosis risk assessment for adolescents diagnosed with autism. Autism, 24(4), 834–850. 10.1177/1362361320909173 32429816

[bjc70020-bib-0117] Wouters, S. G. M. , & Spek, A. A. (2011). The use of the autism‐spectrum quotient in differentiating high‐functioning adults with autism, adults with schizophrenia and a neurotypical adult control group. Research in Autism Spectrum Disorders, 5(3), 1169–1175. 10.1016/j.rasd.2011.01.002

[bjc70020-bib-0118] Ying, J. , Zhang, M. W. , Sajith, S. G. , Tan, G. M.‐Y. , & Wei, K.‐C. (2023). Misdiagnosis of psychosis and obsessive‐compulsive disorder in a young patient with autism Spectrum disorder. Case Reports in Psychiatry, 2023, 1–5. 10.1155/2023/7705913 PMC9943599 36824478

[bjc70020-bib-0119] Zeidan, J. , Fombonne, E. , Scorah, J. , Ibrahim, A. , Durkin, M. S. , Saxena, S. , Yusuf, A. , Shih, A. , & Elsabbagh, M. (2022). Global prevalence of autism: A systematic review update. Autism Research, 15(5), 778–790. 10.1002/aur.2696 35238171 PMC9310578

[bjc70020-bib-0120] Zhang, L. , Sun, Y. , Chen, F. , Wu, D. , Tang, J. , Han, X. , Ye, J. , & Wang, K. (2016). Psychometric properties of the autism‐Spectrum quotient in both clinical and non‐clinical samples: Chinese version for mainland China. BMC Psychiatry, 16, 213. 10.1186/s12888-016-0915-5 27388335 PMC4936315

[bjc70020-bib-0121] Zheng, Z. , Zheng, P. , & Zou, X. (2018). Association between schizophrenia and autism spectrum disorder: A systematic review and meta‐analysis. Autism Research, 11(8), 1110–1119. 10.1002/aur.1977 30284394

[bjc70020-bib-0122] Zhou, H. , Wang, Y. , Zhang, R. , Cheung, E. F. C. , Pantelis, C. , & Chan, R. C. K. (2021). Neural correlates of audiovisual temporal binding window in individuals with schizotypal and autistic traits: Evidence from resting‐state functional connectivity. Autism Research, 14(4), 668–680. 10.1002/aur.2456 33314710

[bjc70020-bib-0123] Zhou, H. , Yang, H. , Gong, J. , Cheung, E. F. C. , Gooding, D. C. , Park, S. , & Chan, R. C. K. (2019). Revisiting the overlap between autistic and schizotypal traits in the non‐clinical population using meta‐analysis and network analysis. Schizophrenia Research, 212, 6–14. 10.1016/j.schres.2019.07.050 31387828

[bjc70020-bib-0124] Ziermans, T. B. , Schirmbeck, F. , Oosterwijk, F. , Geurts, H. M. , & de Haan, L. (2021). Autistic traits in psychotic disorders: Prevalence, familial risk, and impact on social functioning. Psychological Medicine, 51(10), 1704–1713. 10.1017/S0033291720000458 32151297 PMC8327624

