# Supplementary material for: The association between autism and psychosis and the tools used to measure it: An updated systematic review and meta‐analysis
Source: Br J Clin Psychol. 2025 Dec 16;65(2):374–400. doi: 10.1111/bjc.70020 (PMC13159781; doi:10.1111/bjc.70020)
Supplement: Supplementary file 1 — Data S1. [file BJC-65-374-s001.docx]

**Supplementary document**

**Table 1.**

*AXIS Critical Appraisal of Cross-Sectional Studies tool - List of items*

| **AXIS Items** |
| --- |
| A. Introduction |
| 1. Were the aims/objectives of the study clear? |
| B. Methods |
| 2. Was the study design appropriate for the stated aim(s)? |
| 3. Was the sample size justified? |
| 4. Was the target/reference population clearly defined? (Is it clear who the research was about?) |
| 5. Was the sample frame taken from an appropriate population base so that it closely represented the target/reference population under investigation? |
| 6. Was the selection process likely to select subjects/participants that were representative of the target/reference population under investigation? |
| 7. Were measures undertaken to address and categorize non-responders? |
| 8. Were the risk factor and outcome variables measured appropriate to the aims of the study? |
| 9. Were the risk factor and outcome variables measured correctly using instruments/measurements that had been trialed, piloted, or published previously? |
| 10. Is it clear what was used to determine statistical significance and/or precision estimates? (e.g., p-values, CIs) |
| 11. Were the methods (including statistical methods) sufficiently described to enable them to be repeated? |
| C. Results |
| 12. Were the basic data adequately described? |
| 13. Does the response rate raise concerns about non-response bias? |
| 14. If appropriate, was information about non-responders described? |
| 15. Were the results internally consistent? |
| 16. Were the results presented for all the analyses described in the methods? |
| D. Discussion |
| 17. Were the authors’ discussions and conclusions justified by the results? |
| 18. Were the limitations of the study discussed? |
| E. Other |
| 19. Were there any funding or conflicts of interest reported? |
| 20. Was ethical approval or consent of participants reported? |

**Figure 1.**


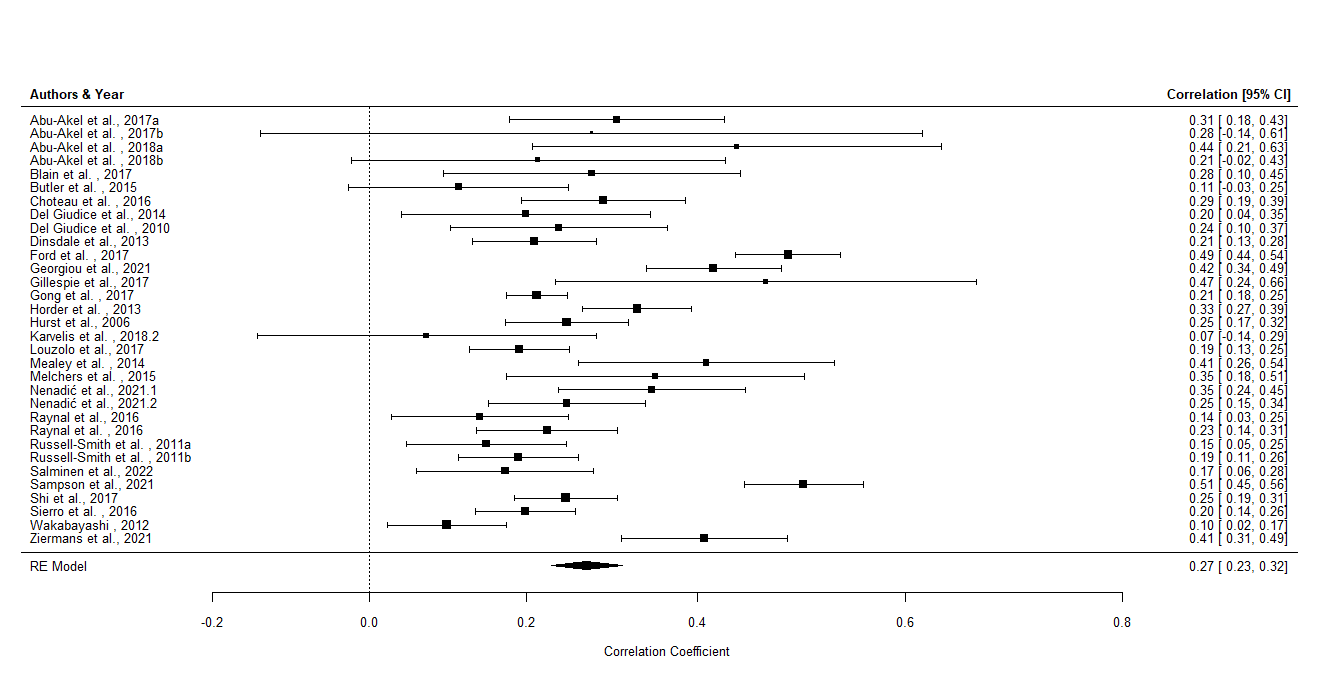
*Forest Plot of Studies Examining the Association between Autistic and Positive Psychotic Symptoms*

**Figure 2.**


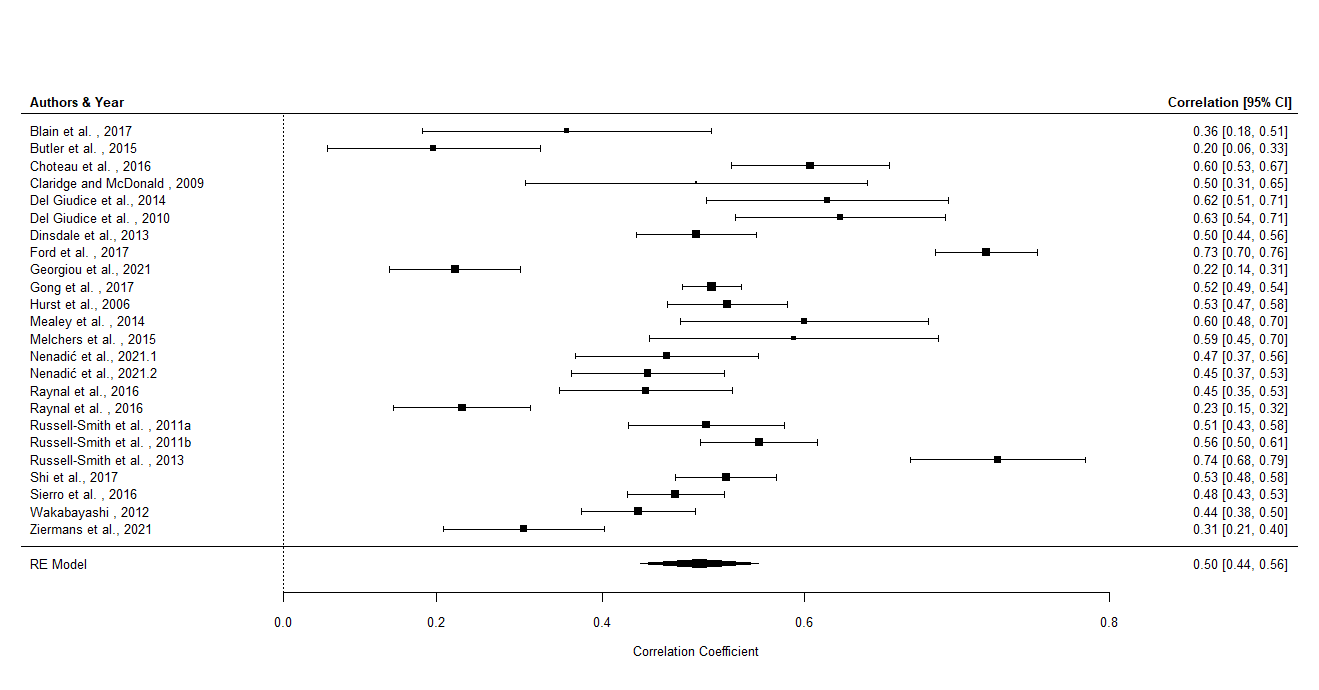
*Forest Plot of Studies Examining the Association between Autistic and Negative Psychotic Symptoms*

**Figure 3.**


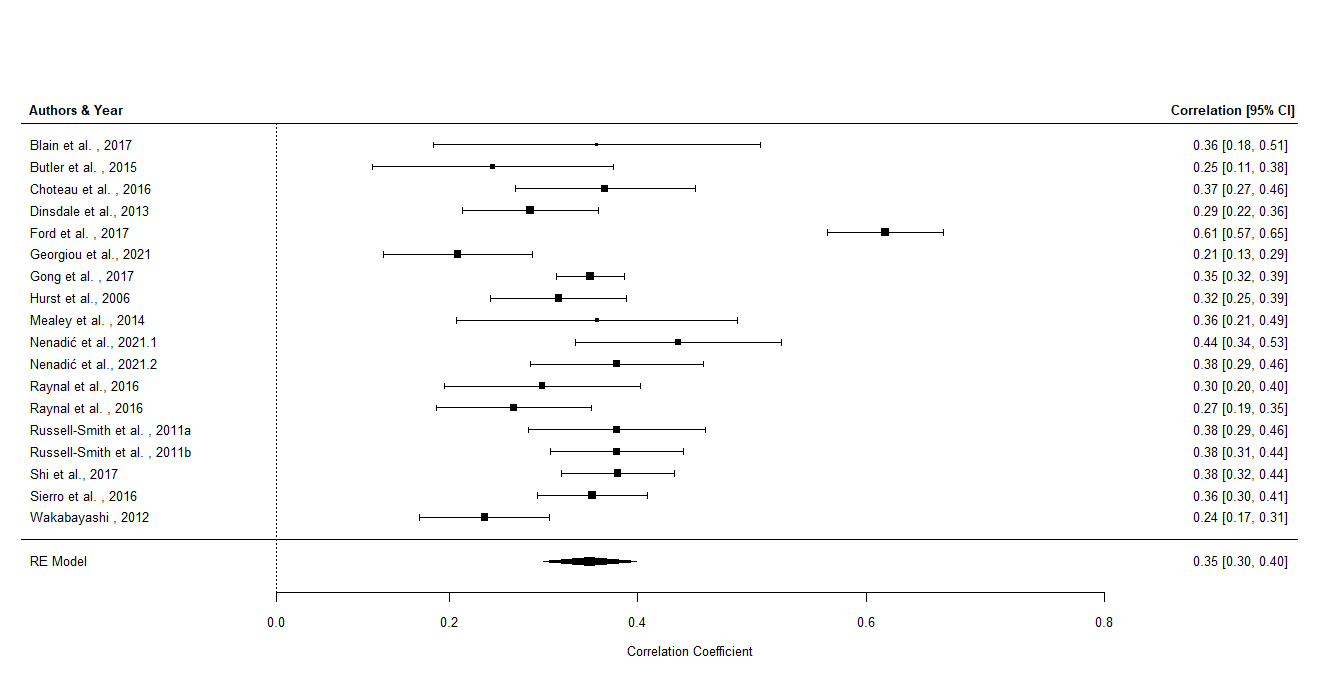
*Forest Plot of Studies Examining the Association between Autistic and Disorganised Psychotic Symptoms*

**Figure 4.**

*Baujat Plot of Studies Examining the Association between Autistic and Overall Psychotic Symptoms*


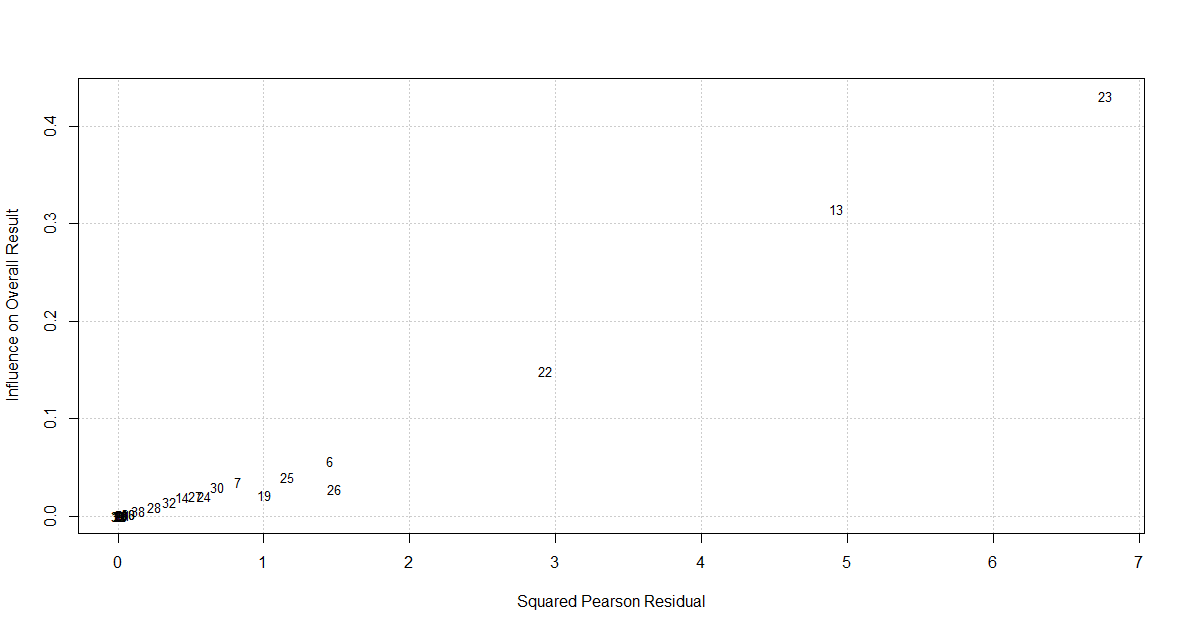


*Note:* Studies 13, 21, and 23, illustrated above, are Ford et al. (2017), Mamah et al. (2021), and Martinez et al. (2021), respectively.

**Figure 5.**

*Baujat Plot of Studies Examining the Association between Autistic and Positive Psychotic Symptoms*


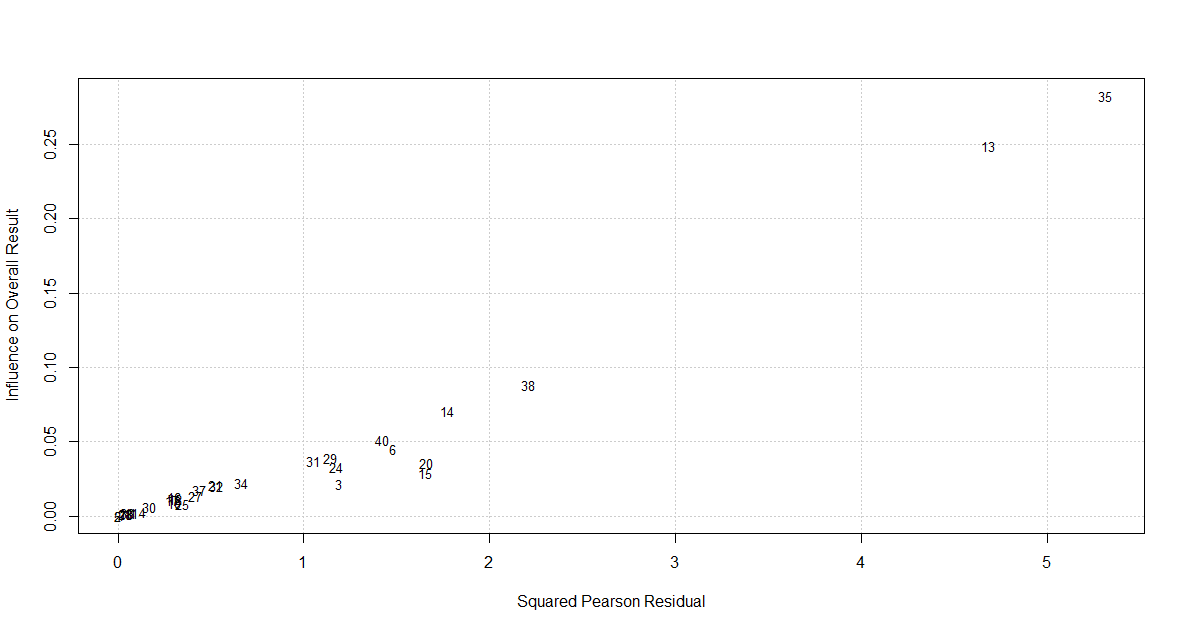


*Note:* Studies 13 and 35, illustrated above, are Ford et al. (2017) and Sampson et al. (2021), respectively.

**Figure 6.**

*Baujat Plot of Studies Examining the Association between Autistic and Negative Psychotic Symptoms*


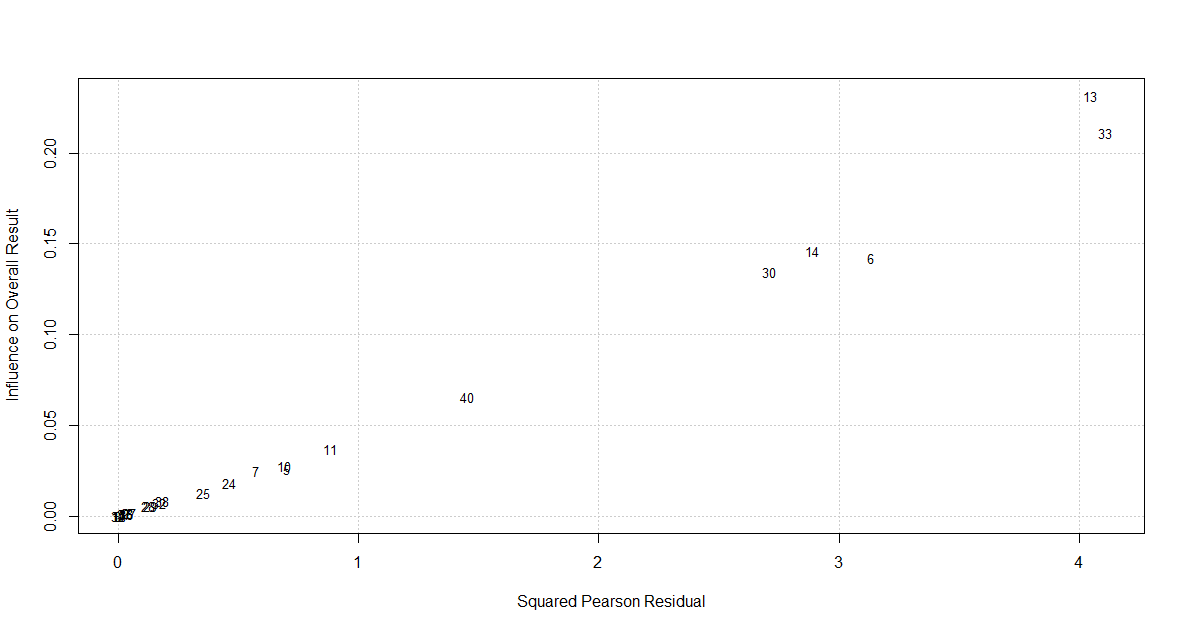


*Note:* Studies 6, 13, 14, 30, and 33, illustrated above, are Butler et al. (2015), Ford et al. (2017), Georgiou et al. (2021), Raynal et al. (2016), and Russell-Smith et al. (2013), respectively.

**Figure 7.**

*Baujat Plot of Studies Examining the Association between Autistic and Disorganised Psychotic Symptoms*


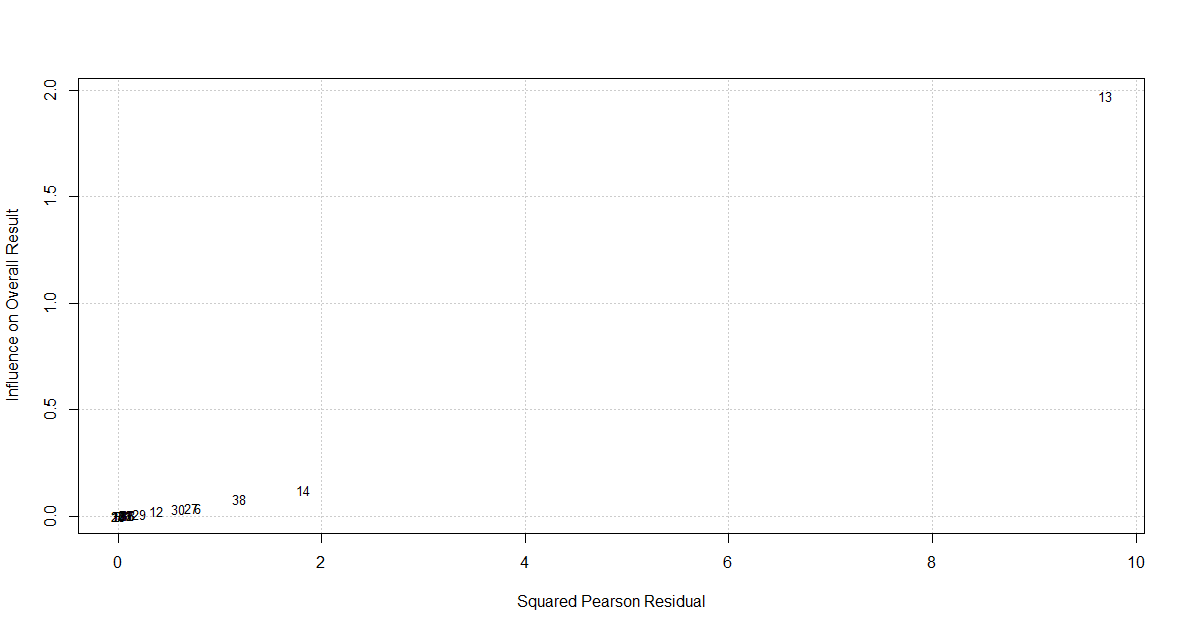


*Note:* Study 13, illustrated above, is Ford et al. (2017).

**Figure 8.**

*Baujat Plot for Odds Ratio Analysis*

**
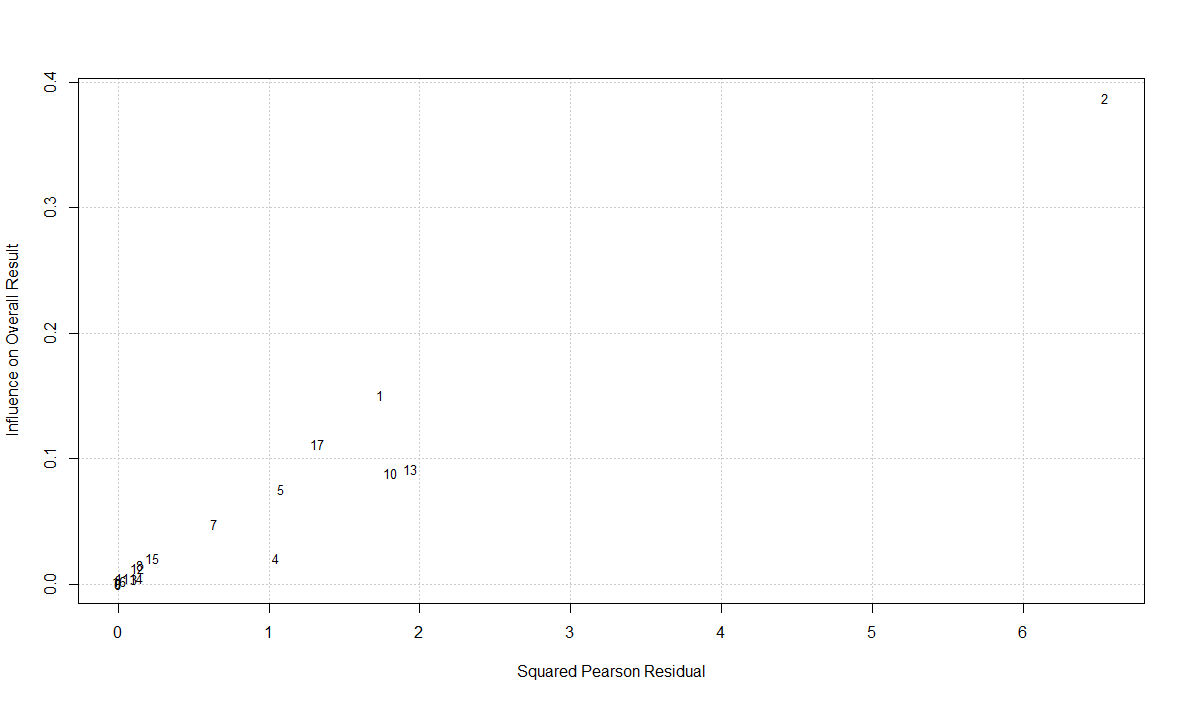
**

*Note:* Study 2, illustrated above, is Chen et al. (2015).

**Figure 9.**

*Baujat Plot for Standardised Mean Difference Analysis*

**
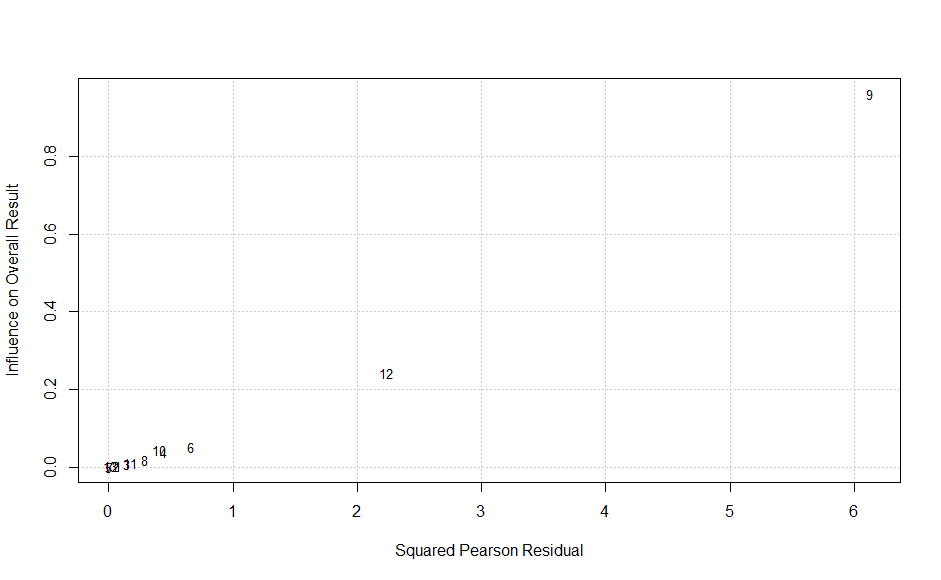
**

*Note:* Study 9, illustrated above, is Suen et al. (2024).
